# Efficient electrochemical production of glucaric acid and H_2_ via glucose electrolysis

**DOI:** 10.1038/s41467-019-14157-3

**Published:** 2020-01-14

**Authors:** Wu-Jun Liu, Zhuoran Xu, Dongting Zhao, Xiao-Qiang Pan, Hong-Chao Li, Xiao Hu, Zhi-Yong Fan, Wei-Kang Wang, Guo-Hua Zhao, Song Jin, George W. Huber, Han-Qing Yu

**Affiliations:** 1 CAS Key Laboratory of Urban Pollutant Conversion, Department of Applied Chemistry, University of Science & Technology of China, Hefei, 230026 China; 2 Department of Chemical and Biological Engineering, University of Wisconsin-Madison, Madison, WI 53706 USA; 3 School of Chemical Science and Engineering, Tongji University, Shanghai, China; 4 Department of Chemistry, University of Wisconsin-Madison, Madison, WI 53706 USA

**Keywords:** Energy, Renewable energy, Electrocatalysis

## Abstract

Glucose electrolysis offers a prospect of value-added glucaric acid synthesis and energy-saving hydrogen production from the biomass-based platform molecules. Here we report that nanostructured NiFe oxide (NiFeO_x_) and nitride (NiFeN_x_) catalysts, synthesized from NiFe layered double hydroxide nanosheet arrays on three-dimensional Ni foams, demonstrate a high activity and selectivity towards anodic glucose oxidation. The electrolytic cell assembled with these two catalysts can deliver 100 mA cm^−2^ at 1.39 V. A faradaic efficiency of 87% and glucaric acid yield of 83% are obtained from the glucose electrolysis, which takes place via a guluronic acid pathway evidenced by in-situ infrared spectroscopy. A rigorous process model combined with a techno-economic analysis shows that the electrochemical reduction of glucose produces glucaric acid at a 54% lower cost than the current chemical approach. This work suggests that glucose electrolysis is an energy-saving and cost-effective approach for H_2_ production and biomass valorization.

## Abstract (web summary)

Renewable biomass conversion may afford high-value products from common materials, but catalysts usually require expensive metals and exhibit poor selectivities. Here, authors employ nickel-iron oxide and nitride electrocatalysts to produce H_2_ and to convert glucose to glucaric acid selectively.

## Introduction

Biomass conversion into commodity chemicals is a promising strategy to reduce society’s dependence on fossil fuel resource^1–6^. Glucose, one of the most abundant biomass-based compounds, can be converted into various commodity chemicals like 5-hydroxymethylfurfural, sorbitol, gluconic acid (GNA), and glucaric acid (GRA)^2,7,8^. GRA is recognized as a “top value added compound” produced from biomass^9^, because it is a key intermediate for the production of biodegradable polymers, biodegradable detergents, and metal complexation agents^10,11^. Moreover, GRA and its derivatives (e.g., GRA-Ca, GRA-1,4-lactone) can also be used for healthcare purposes such as cancer chemotherapy and cholesterol reduction^12–14^. According to a market report by Grand View Research, Inc.^15^, the global GRA market size in 2016 was about USD 550.4 million and is expected to reach USD 1.30 billion by 2025.

GRA is currently produced from either chemical oxidation^16–18^ or microbial fermentation, and the former is the main industrial process for GRA production^19–21^. For example, Rivertop Renewables Inc. (Montana, USA) has developed a catalytic oxidation process to produce GRA from glucose with an annual output of 25k tons^22^. Chemical oxidation is performed either by stoichiometric oxidation of glucose with HNO_3_ in the absence of catalysts^23,24^, or by catalytic oxidation of glucose with O_2_ (air) in the presence of noble metal (e.g., Au, Pt, Pd, and Ru)-based catalysts at 45−120 °C^25–28^. In chemical oxidation, various byproducts such as GNA, glucuronic acid, and other organic acids are formed. For example, Qi et al.^29^ investigated glucose oxidation over an Au-based catalyst with O_2_ (0.3 MPa) at 120 °C, with a GNA yield of 92% and a GRA yield of less than 5%. Jin et al.^30^ reported oxidation of glucose to GRA with a yield of 45% over bimetallic Pt-Cu catalysts at 45 °C and 0.1 MPa O_2_. Conventional catalytic oxidation of glucose to GRA has several shortcomings: (1) a large amount of toxic oxidant is required (more than double the stoichiometric ratio); (2) a low selectivity to GRA (the GRA selectivity is reported to be less than 60%); and (3) generation of various byproducts (e.g., 2,3-dihydroxysuccinic acid, 2,3-dihydroxy-4-oxobutanoic acid, and oxalic acid) with similar chemical properties; and (4) use of high pressure O_2_ (or air and HNO_3_). Avoiding the use of high pressure O_2_ (or air) reduces the use of high pressure vessels and safety risks. Microbial fermentation does not consume as many raw materials and operates at less severe operating conditions compared to chemical oxidation. However, fermentation also suffers from various disadvantages such as long fermentation time (more than 2 days), low selectivity (GRA yield < 20%), and difficulty in product separation (large amounts of microbial biomass and hundreds of byproducts with similar properties are co-produced)^31^.

Electrochemical oxidation involves electron transfer in the reactions (Scheme 2) and eliminates the use of high pressure O_2_ or hazardous chemical oxidants. The electrochemical oxidation process can be operated under mild conditions, and production of other byproducts can be easily suppressed via tuning the electrode potential, leading to a high GRA selectivity. Electrochemical oxidation could be practiced commercially at small scales in distributed areas and becomes cheaper as the price of renewable electricity produced continues to decline^32^. The electrochemical oxidation process is particularly suitable for GRA production, as GRA is produced on a smaller scale, and therefore cannot take advantage of improved economics due to large economies of scale that larger bulk chemicals have. Glucose electrolysis to GRA provides H_2_ gas as a product stream as hydrogen evolution reaction (HER) occurs at the cathode and the oxidation of glucose occurs at the anode. This reaction also has a lower standard redox potential (0.05 V) than conventional water electrolysis (1.23 V)^33,34^.

Although the anodic oxidation of glucose can be achieved efficiently with the noble metal (e.g., Pt, Ru, Rh, and Pd)-based catalysts^35,36^, their scarcity and high costs motivate researchers to seek abundant and inexpensive alternative catalysts. Recent research on electrochemical oxidation has developed a series of earth-abundant transition metal-based catalysts with high catalytic activity and stability towards the electro-oxidation of various organic compounds, such as Co_3_O_4_ nanosheets for ethanol electro-oxidation^37^, Ni–Mo-based nanostructures for urea electro-oxidation^38^, Co-Cu-based nanostructures for benzyl alcohol electro-oxidation^39^, and Ni-Fe LDH nanosheets for 5-Hydroxymethylfurfural electro-oxidation^40^.

In previous studies of electrochemical oxidation of glucose, GRA yields were reported to be less than 40%^41^, although Ibert et al.^42^ reported that 2, 2, 6, 6-tetramethyl-1-piperidinyloxy (TEMPO)-mediated electro-oxidation of glucose could obtain GRA with a yield of 85%. However, the high-cost and difficulty in TEMPO separation and recycle may hinder this technology’s industrial application. It has been a challenge to produce GRA with a high yield in a cost-effective way.

In this work, we synthesize the NiFe oxides (NiFeO_*x*_) and NiFe nitrides (NiFeN_*x*_) using the NiFe LDH nanosheet arrays as a precursor material, and investigate their electrocatalytic performance toward anodic glucose oxidation and cathodic HER using various electrochemical approaches. These catalysts show a high activity and selectivity towards anodic glucose oxidation and cathodic hydrogen evolution respectively.

## Results

### Synthesis and characteristics of the catalyst materials

**Fig. 1:**
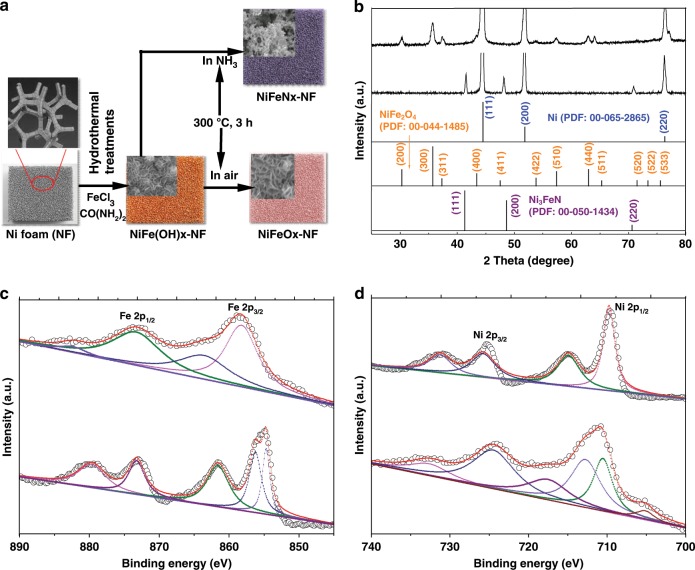
Synthesis and structure characterization of the catalysts. **a** Schematic illustration for the synthesis of NiFeN_*x*_-NF and NiFeO_*x*_-NF catalysts. **b** XRD patterns of the NiFeO_*x*_-NF and NiFeN_*x*_-NF catalysts in comparison with standard XRD patterns. **c** XPS Fe 2p spectra and **d** Ni 2p spectra of the NiFeO_*x*_-NF and NiFeN_*x*_-NF catalysts.

The structural and composition characterizations of the as-synthesized catalysts are presented in Supplementary Method 1. Figure 1a is a schematic for the synthesis of Ni-Fe-OH catalyst. A commercially available nickel foam (NF) with open fibrous structure was used to confer the 3D structure of the electrode and provide the Ni source for the NiFe(OH)_*x*_. The NiFe(OH)_*x*_ precursor grew on the NF through a hydrothermal reaction of FeCl_3_ and CO(NH_2_)_2_ (urea) in a mixed solution. Urea was added to the solution to increase the pH, which resulted in the controlled hydrolysis of the Ni and Fe metal ions^40,43^. The X-ray diffraction (XRD) pattern (Supplementary Fig. 1) of the resulting solid indicates the formation of hydrotalcite-like NiFe layer double hydroxides, Ni(OH)_2_, and FeOOH crystalline phases.

The NiFe(OH)_*x*_ precursor was then heated at 300 °C for 3 h under air flow to form Ni-Fe oxides (NiFeO_*x*_). The XRD pattern (Fig. 1b) shows that the diffraction peaks matched those of NiFe_2_O_4_ (PDF: 00-044-1485). The NiFe(OH)_*x*_ precursor was also heated in an NH_3_ flow to obtain NiFe nitrides (NiFeN_*x*_), and the diffraction peaks of this product matched those of Ni_3_FeN (PDF: 00-050-1434). Three large diffraction peaks of Ni metal are in both XRD patterns, which are the Ni foam.

The surface chemistry and composition of the catalysts were probed with X-ray photoelectron spectroscopy (XPS). The Fe 2p spectra of NiFeO_*x*_ and NiFeN_*x*_ are shown in Fig. 1c. Two peaks located at binding energy of 725.2 and 711.7 eV of NiFeO_*x*_ spectrum are assigned to the Fe 2p_1/2_ and Fe 2p_3/2_, respectively, with a difference of 13.5 eV, implying that the Fe in the NiFeO_*x*_ was in its tri-valent oxidation state (Fe(III))^44^. The Fe 2p_3/2_ peak in the NiFeN_*x*_ was deconvoluted into two peaks, which are assigned to Fe-O species (711.9 eV) and Fe-N species (710.5 eV), respectively^45,46^. The Ni 2p spectra of NiFeO_*x*_ and NiFeN_*x*_ are presented in Fig. 1d. Two prominent peaks located at 856 and 874 eV of NiFeO_*x*_ spectrum were attributed to the 2p_3/2_ peaks from Ni and 2p_1/2_ peaks in Ni-O species, respectively. The Ni 2p_3/2_ in the NiFeN_*x*_ was deconvoluted into two peaks attributed to the Ni-O (856.1 eV) and Ni-N (854.7 eV) species, respectively. For comparison, the Fe 2p and Ni 2p XPS spectra of the NiFe(OH)_*x*_ precursor in Supplementary Fig. 2 show that the Ni 2p spectrum of NiFe(OH)_*x*_ was almost the same as that of NiFeO_*x*_, suggesting that the chemical state of Ni was unchanged during the heat treatment in air. The Fe 2p 3/2 branch in NiFe(OH)_*x*_ could be deconvoluted into two peaks, which are attributed to the Fe(II) (710.4 eV) and Fe(III) (712.8 eV) species, respectively. The Fe(II) species cannot be found in the NiFeO_*x*_ sample, confirming that heat treatment transformed Fe(II) into Fe(III). In comparison with NiFe(OH)_*x*_, the oxidation states of Ni and Fe in the NiFeN_*x*_ sample were reduced to lower valence.

**Fig. 2:**
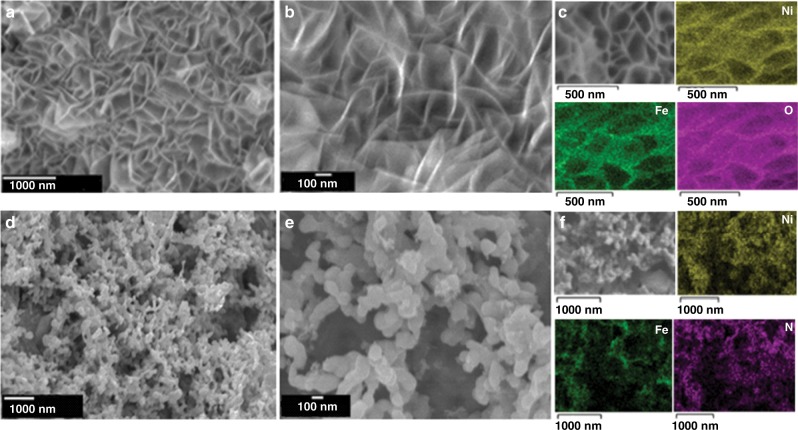
Morphology and elemental compositions of the electrocatalysts. SEM images of the NiFeO_*x*_-NF catalyst in low (**a**) and high (**b**) magnification; **c** EDS elemental mapping of the NiFeO_*x*_-NF catalyst; SEM images of the NiFeN_*x*_-NF catalyst in low (**d**) and high (**e**) magnification; and **f** EDS elemental mapping of the NiFeN_*x*_-NF catalyst.

The morphology of the NiFe(OH)_*x*_, NiFeO_*x*_, and NiFeN_*x*_ samples were examined with scanning electron microscopy (SEM). Typical SEM images of NiFe(OH)_*x*_ (Supplementary Fig. 3) show that the as-synthesized hydroxides consisted of nanosheets aligned vertically on the NF covering the entire substrate. Energy dispersive X-ray spectroscopy (EDS) elemental mapping results (Supplementary Fig. 4) indicate that the Ni, Fe, and O elements were distributed evenly on the NF. After calcination, the obtained NiFeO_*x*_ product retained the nanosheet morphology of the hydroxide precursor (Fig. 2a, b). The EDS results also confirm that the Ni, Fe, and O elements were distributed homogenously (Fig. 2c). However, after the heat treatment in NH_3_, the produced NiFeN_*x*_ material did not maintain the morphology of the hydroxide precursor, but consisted of irregularly shaped interconnected particles with micropores and mesopores (Fig. 2d, e). The EDS elemental mapping (Fig. 2f) confirms the homogenous distribution of the Fe, Ni, and N in the NiFeN_*x*_ sample, and some O element was also found, which might be due to the incomplete nitridation of the NiFe(OH)_*x*_.

### Electrocatalytic glucose oxidation

**Fig. 3:**
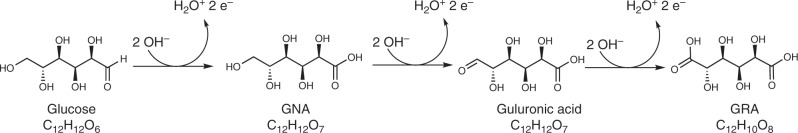
Possible reaction pathway from glucose to GRA. Schematic illustration of the possible pathway for the electrochemical oxidation of glucose to GNA and GRA.

**Fig. 4:**
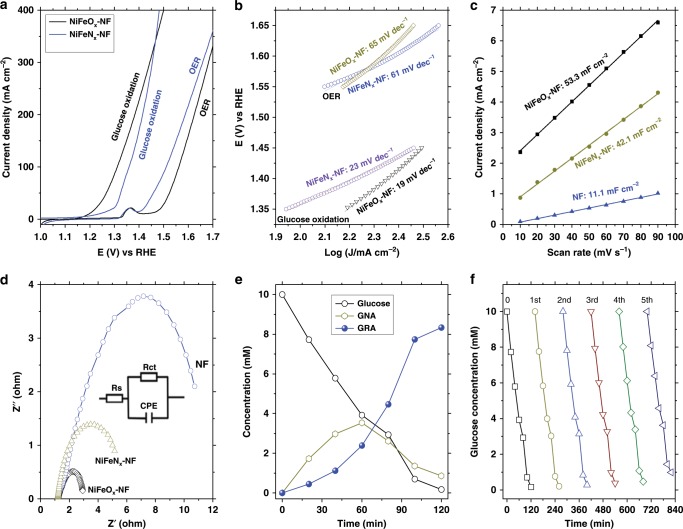
Anodic glucose oxidation. **a** LSV profiles of the NiFeO_*x*_-NF and NiFeN_*x*_-NF catalysts for glucose oxidation and OER (scan rate of 5 mV s^−1^; electrolyte: 1 M KOH; glucose concentration 100 mM). **b** Corresponding Tafel plots. **c** Capacitive current densities of different electrodes for glucose oxidation as a function of scan rate. **d** Nyquist plots (taken at 1.3 V vs. RHE) of different electrodes for glucose oxidation. **e** Concentration of glucose and oxidation products as a function of time for chronoamperometric tests at 1.30 V vs. RHE. **f** Glucose concentration changes in five successive cycles.

The glucose oxidation involves two steps: (1) the oxidation of glucose into GNA, which involves two electrons, and (2) the oxidation of GNA to GRA, which involves four electrons (Fig. 3). Oxygen evolution reaction (OER) is the main undesired competing reaction^47,48^. The linear sweep voltammetry (LSV) profiles and the corresponding parameters for the glucose (100 mM) oxidation and OER with the NiFeO_*x*_-NF and NiFeN_*x*_-NF electrodes in 1 M KOH solution (pH = 13.9) are presented in Fig. 4a, Table 1, and Supplementary Table 5. NiFeO_*x*_-NF presented a current density of 87.6 mA cm^−2^ at a potential of 1.30 V (vs. reversible hydrogen electrode, RHE) for glucose oxidation with a TOF value of 0.16 s^−1^ (entry 5, Table 1). The NiFeN_*x*_-NF electrode displayed a lower current density (22.1 mA cm^−2^) and with a TOF value of only 0.04 s^−1^ (entry 6, Table 1), confirming that NiFeO_*x*_ was a more active catalyst for glucose oxidation than NiFeN_*x*_. However, for the OER, NiFeN_*x*_ (entry 2, Table 1) was more efficient than NiFeO_*x*_ (entry 1, Table 1). Both the current densities and TOF values for glucose oxidation were much lower than the current densities and TOF for OER. NiFeN_*x*_ oxidized into high surface-area metal oxides/hydroxides in the oxidative environment of OER reaction (Supplementary Fig. 5) and also likely in the glucose oxidation reactions^49^. The Tafel slopes (Fig. 4b) for glucose oxidation with NiFeO_*x*_-NF and NiFeN_*x*_-NF electrodes were calculated as 19 and 23 mV dec^−1^, respectively, which were much lower than the Tafel slopes for OER with those two electrodes. The Tafel slope is closely related to the electron transfer rate, and a smaller Tafel slope means a more rapid electron transfer rate and more favorable catalytic reaction kinetics^50^. The lower Tafel slope of the anodic glucose oxidation confirms that more rapid electron transfer occurs in the oxidation of CHO group to COOH in C1 position (two-electron transfer) and the oxidation of CH_2_OH group to COOH in C6 position (four-electron transfer) than the cleavage of O−H bonds in H_2_O molecule to produce O_2_. The low value of the Tafel slope also implies a lower adsorption potential of glucose to the electrode^51^ and the intimate contact between the electrode catalysts and electrolyte^52^.

**Table 1:** Electrochemical anodic glucose oxidation and OER in 1 M KOH.

Entry	Catalyst	C_glucose_ (mM)	J (mA cm^−2^) (*E* = 1.30 V)	TOF (s^−1^) (*E* = 1.30 V)	Potential applied (V)	Reaction time (h)	Glucose conversion (%)	*Y*_(GNA)_ (%)	*Y*_(GRA)_ (%)	*Y*_(GNA+GRA)_ (%)	FE (%)
1	NiFeO_*x*_-NF	0	2.61	1.6 × 10^−3^	—	—	—	—	—	—	—
2	NiFeN_*x*_-NF	0	0.90	4.7 × 10^−3^	—	—	—	—	—	—	—
3	NiFeO_*x*_-NF	10	17.7	0.03	1.30	2	98.3	8.6	83.3	91.9	87
4	NiFeO_*x*_-NF	50	61.5	0.11	1.30	10	93.1	11.2	75.3	86.5	79
5	NiFeO_*x*_-NF	100	87.6	0.16	1.30	18	90.6	12.6	71.2	83.8	73
6	NiFeN_*x*_-NF	100	22.1	0.04	1.30	18	92.7	19.6	63.8	83.4	68
7	NiFe(OH)_*x*_-NF	100	79.2	0.09	1.30	18	88.6	21.3	56.9	78.2	64
8	NF	100	20.3	1.7 × 10^−3^	1.30	18	59.2	37.3	17.2	54.5	67
9	Pt-C//NF	100	49.3	0.04	1.30	18	67.3	41.9	20.2	62.2	71
10	RuO_2_//NF	100	83.8	9.4 × 10^−3^	1.30	18	90.1	52.2	27.7	79.9	77

It should be noted that unlike many previous reports, in which the electrode materials with high OER performance usually have high catalytic performance for oxidation of organic compounds^37,38,40^, the NiFeO_*x*_-NF electrode had a lower OER performance compared to NiFeN_*x*_-NF, but possessed a higher glucose oxidation performance in a low potential region. To probe why this phenomenon occurred, the relative electrochemically active surface areas (ECSA) in glucose oxidation with the electrodes were compared by extracting their double-layer capacitances using cyclic voltammetry (CV). The CV profiles (Supplementary Fig. 6) were collected in the non-Faradaic potential region (0.925−1.0 V), in which only double-layer capacitance is accounted for the current response. The capacitance for NiFeO_*x*_-NF was calculated to be 53.3 mF cm^−2^ based on the CV results, higher than those of NiFeN_*x*_-NF (42.1 mF cm^−2^) and only NF (11.1 mF cm^−2^) (Fig. 4c). Thus, the NiFeO_*x*_-NF electrode had a larger ECSA than the NiFeN_*x*_-NF electrode. This indicates that the higher catalytic activity of NiFeO_*x*_-NF was likely due to the higher number of catalytic active sites, and the in situ generated surface Ni-Fe oxyhydroxides (FeOOH and NiOOH) could be the catalytic active sites of NiFeO_*x*_-NF electrode for both glucose oxidation and OER^53,54^. For example, the formation of Ni oxyhydroxides was confirmed by their polarization curves of OER (Fig. 4a), in which a small peak located at *E* = 1.36 V could be attributed to the oxidation of Ni(II) into NiOOH: (NiO + OH^−^ → NiOOH + e−). The formation of FeOOH could not be confirmed by the polarization curves as it does not experience a valence variation in the electrochemical process, but the XPS spectra of NiFeO_*x*_-NF after glucose oxidation (Supplementary Fig. 5) confirm the formation of NiOOH and FeOOH species. We have also carried out control experiments to verify that the Ni-Fe oxyhydroxides are the catalytic active sites. The NiFeO_*x*_-NF and NiFeN_*x*_-NF catalysts were treated with H_2_O_2_ in 1 M of KOH solution to produce more NiOOH and FeOOH species. These treated catalysts were then used as anodic catalysts for both glucose oxidation and OER. The LSV profiles of these materials shown in Supplementary Fig. 7 indicate that after the H_2_O_2_ treatments, both NiFeO_*x*_-NF and NiFeN_*x*_-NF catalysts exhibited higher catalytic activities for OER (Supplementary Fig. 7a) and glucose oxidation (Supplementary Fig. 7b). Such improvements in the catalytic activities should be attributed to the higher activity of the Ni-Fe oxyhydroxides sites.

Electrochemical impedance spectroscopy (EIS) was used to investigate the kinetics of the electrode materials. An equivalent circuit consisting of a series resistance (*R*_s_), a charge-transfer resistance (*R*_ct_), and a constant phase element (CPE) was constructed. As shown in Fig. 4d, the NiFeO_*x*_-NF electrode displayed a smaller charge-transfer resistance (*R*_ct_) of about 1.7 Ohm, in contrast to the NiFeO_*x*_-NF (3.8 Ohm) and raw nickel foam electrodes (9.5 Ohm). The small *R*_ct_ means favorable electron transport rate and catalytic kinetics, resulting in a small Tafel slope^55^. Meanwhile, the EIS profiles also show that the NiFeO_*x*_-NF and NiFeN_*x*_-NF electrodes had small *R*_s_ values (1.21 and 1.26 Ohm, respectively), revealing good electrical contacts between the catalysts and the nickel foam substrate. This was attributed to the formation of NiFeO_*x*_ and NiFeN_*x*_ catalysts via direct reactions of Ni foam, leading to strong adhesion to the Ni foam substrate.

We also investigated the catalytic performance of other catalysts, including NiFe(OH)_*x*_-NF, NF, as well as the benchmark Pt/C and RuO_2_ catalysts, for glucose oxidation (Supplementary Fig. 8; also see entries 7−10, Table 1). Among all the examined catalysts, the NiFeO_*x*_-NF electrode demonstrated the highest catalytic activity, with the lowest *E*_onset_ and *E*_*j*_ = 100 mA cm^−2^ values. The highest catalytic activity of the NiFeO_*x*_-NF electrode could be attributed to the following two reasons: (1) it possessed the highest number of catalytic active sites as indicated from the ECSA (Supplementary Fig. 9); and (2) it had the lowest charge-transfer resistance (Supplementary Fig. 10), which means a high electron transport rate and rapid catalytic kinetics. The rapid catalytic kinetics of the NiFeO_*x*_-NF electrode can also be revealed by its lowest Tafel slope (Fig. 4b).

The effect of glucose concentration on glucose oxidation was studied with the NiFeO_*x*_-NF electrode (Table 1). Supplementary Fig. 11a shows the LSV profiles of the NiFeO_*x*_-NF for glucose oxidation at various concentrations. The current density at *E* = 1.25 V had a linear relationship with the glucose concentration for 0−150 mM glucose concentration (Supplementary Fig. 11b). The current density did not change with a further increase in glucose concentration (150−500 mM). The electrochemical glucose oxidation followed the first-order reaction kinetics at low glucose concentrations, but changed into the zero-order reaction kinetics after the glucose concentration exceeding 150 mM. A similar result was reported for the glucose oxidation on RuO_2_ electrodes in 1 M of NaOH solution^56^.

The chronoamperometric measurements of glucose oxidation were conducted at a constant potential of 1.30 V, and the concentrations of glucose and its oxidation products (GNA and GRA) in chronoamperometric tests were monitored with HPLC as detailed in Supplementary Method 2 and Supplementary Fig. 12. The concentrations of the products and reactant as a function of reaction time are shown in Fig. 4e (initial concentration: 10 mM). These results indicate that GNA was the initial product, which was then converted into GRA. The glucose conversion after 120-min reaction was 98.3%, with a yield of GNA plus GRA of 91.9%, and a Faradaic efficiency (FE) for GNA plus GRA production of 87% (entry 3, Table 1). We also studied the potential glucose decomposition in an alkaline aqueous solution on open circuit with ^1^H, ^13^C nuclear magnetic resonance (NMR) and 2D-HSQC NMR (Supplementary Fig. 13). Glucose (10 mmol/L) was not degraded after 24 h in the alkaline solution, suggesting that no significant self-decomposition of glucose occurred under the reaction conditions. This result is consistent with other glucose oxidation studies in alkaline solutions^57,58^.

Five successive cycles of chronoamperometric measurements were conducted to evaluate the stability and durability of the NiFeO_*x*_-NF electrode for glucose oxidation. The conversion of glucose slightly decreased from 98.3 to 91.2% after five cycles (Fig. 4f), and the reaction rates slightly decreased from 1.36 × 10^−4^ to 1.27 × 10^−4^ mmol glucose s^−1^ in these five successive cycles, but the FE values (Supplementary Fig. 14) of each cycle remained almost unchanged. The XRD patterns (Supplementary Fig. 15) of the catalyst after five cycles show that NiFe_2_O_4_ was the main crystalline phase. The XPS spectra of the reused NiFeO_*x*_-NF catalyst (Supplementary Fig. 16) show a new peak at 857.4 eV in the Ni 3d spectrum, which could be attributed to the Ni(III) species, suggesting that some Ni(III) species were formed in the anodic oxidation process. It is reported that the formation of Ni(III) is important for the anodic organic compound oxidation because the higher-valence state of Ni facilitates the adsorption of OH^−^ ions and reduces the energy barrier for the transition of Ni species from lower- to higher-valence states in promoting anodic oxidation reactions^59^. The SEM image (Supplementary Fig. 17) shows that after reuse, the nanosheet structure of the NiFeO_*x*_ kept almost unchanged. The EDS mapping results (Supplementary Fig. 18) of the reused catalyst demonstrate that the Fe and Ni elements were evenly distributed on the NF.

**Fig. 5:**
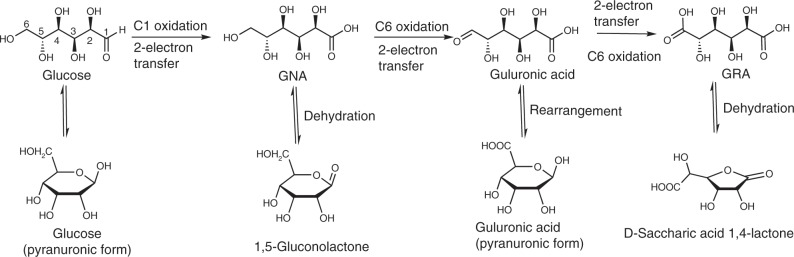
Possible reaction mechanism. Schematic illustration of the reactions occurring in the electrochemical oxidation of glucose to GRA.

**Fig. 6:**
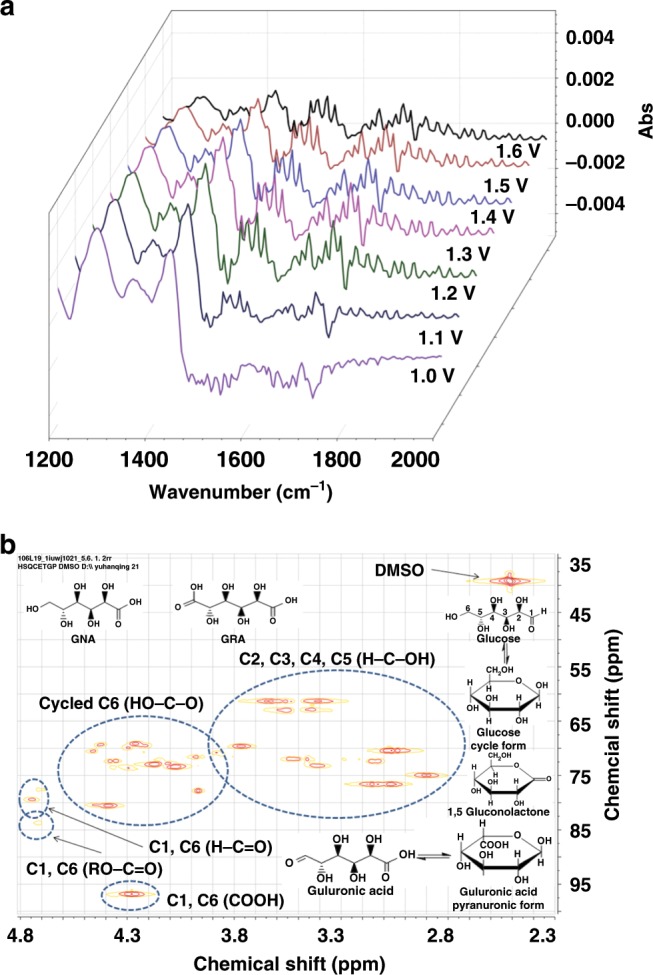
Analysis of the possible products of the glucose electrolysis process. **a** In situ ATR-FTIR spectra collected at a potential ranging from 1.0 to 1.6 V vs. RHE with a step of 100 mV. **b** 2D HSQC NMR spectrum of the reaction solution after 6-h electrolysis (initial glucose concentration: 10 mM, potential: 1.3 V).

To gain some insights into the mechanism of the anodic glucose oxidation over the NiFeO_*x*_-NF catalyst (Fig. 5), in situ ATR-FTIR, 2D-HSQC NMR and LC-MS analysis was performed. The IR spectra collected in the potential step experiments ranging from 1.0 to 1.6 V provides useful information on the glucose oxidation pathways (Fig. 6a). The cleavage of C−C bond did not occur because of the mild conditions of the electrochemical oxidation process. Thus, the possible intermediates and products of glucose oxidation were proposed as follows: GNA and gluconolactone (oxidation of H−C=O group to COOH in C1 position, two-electron transfer), glucuronic acid (oxidation of CH_2_OH group to H−C=O in C6 position, two-electron transfer), and GRA (further oxidation of H−C=O group in C6 position, two-electron transfer). The assignments of IR bands as well as the identification of the reaction intermediates were conducted by comparing with the reference spectra (Supplementary Fig. 19 and Supplementary Table 1). The bands appearing synchronously at wavenumbers of 1573−1506 cm^−1^ and 1483−1431 cm^−1^ were attributed to the asymmetric stretching and symmetric vibrations of O−C−O, respectively, confirming the formation of glucuronic acid (Pyranuronic form). No C−C bond breaking occurred in the electrochemical glucose oxidation process, as no C−C bond cleavage compounds (CO: 1900−2100 cm^−1^ and CO_3_^2−^: 1390−1400 cm^−1^) were observed in the IR spectra. However, the complete oxidation of glucose into GNA and GRA cannot be achieved in the in situ ATR-FTIR reactor due to insufficient reaction time. Another note is that the formation of GNA and GRA could not be differentiated with the FTIR spectra only, as these two compounds have very similar functional group compositions. In addition to the IR spectra, the ^1^H, ^13^C and 2D HSQC NMR as well as LC-MS analyses were also performed. The ^1^H and ^13^C NMR spectra of the reactant and 6-h products from glucose electrolysis are presented in Supplementary Fig. 20. The signals in the chemical shift of 0 and 2.50 ppm could be attributed to the reference TMS (tetramethylsilane) and DMSO-H6 (a main impurity of the deuterated DMSO solvent for NMR), while the signals in the chemical shift of 3−6 ppm could be ascribed to glucose and its oxidation products. A very small signal could be found in the chemical shift of 11.1 ppm, which could be attributed to the H atom in the COOH groups. This small signal does not mean that the COOH content in the product was of a very low level because the intensity of the 1 H NMR signal for COOH is not proportional to the concentration of COOH in the samples. Such a weak H signal is likely to be attributed to the mobility of H in the COOH group^60,61^. Nevertheless, the presence of COOH in the reacted sample was confirmed by the 13C NMR spectra. The C1, C6 (COOH) regions in the 2D HSQC NMR spectrum (Fig. 6b) could be attributed to the GNA, GRA and guluronic acid. To further identify these compounds, we examined the 13C NMR of the standard glucose and its main oxidation products (e.g., GNA, GRA, and guluronic acid). As shown in Supplementary Fig. 21, the presence of glucose was confirmed by the characteristic peaks at chemical shifts of 60.9 and 92.4 ppm, while the presence of GNA could be observed via the characteristic peak at chemical shift of 64.4 ppm and the signal of COOH (*δ* = 175.4 ppm). The presence of GRA could be observed via the characteristic peak at chemical shift of 69.7 ppm and the signal of COOH (*δ* = 175.5 ppm), while the presence of guluronic acid could be observed via the characteristic peak at chemical shift of 97.2 ppm and the signal of COOH (*δ* = 175.7 ppm). The presence of 1,5-gluconolactone could be inferred from the C1, C6 (R−O−C=O) regions in Fig. 6b. The LC-MS analytic results of the reaction solution after 6-h electrolysis (Supplementary Fig. 22) further confirm the presence of GNA, GRA and some intermediates like 1,5-gluconolactone and guluronic acid.

### Cathodic HER

**Fig. 7:**
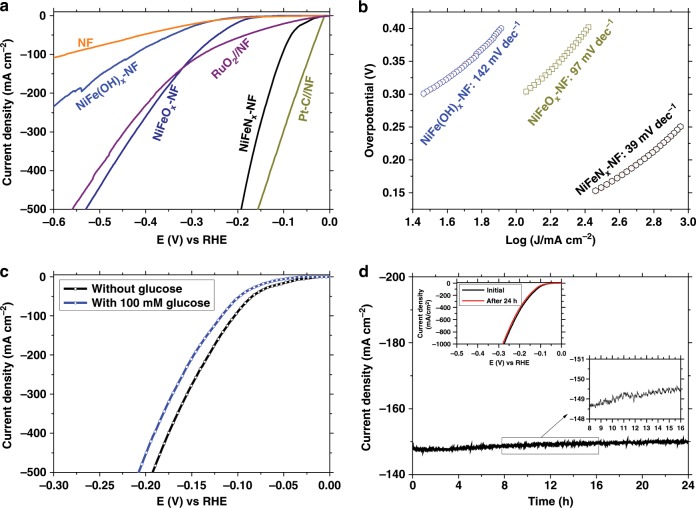
Cathodic HER of the catalyst. **a** LSV profiles of the different electrodes for HER in 1 M KOH electrolyte (scan rate of 5 mV s^−1^). **b** Corresponding Tafel plots. **c** Comparison of LSV profiles of the NiFeN_*x*_-NF catalyst for HER with and without 100 mM of glucose. **d** Chronoamperometric test of the NiFeN_*x*_-NF catalysts for HER with 100 mM of glucose, left inset: LSV profiles of the NiFeN_*x*_-NF catalysts before and after 24-h chronoamperometric test, right inset: amplified region of the chronoamperometric curve.

The cathodic HER under alkaline conditions was also investigated using the Ni-Fe-OH-based electrodes together with other electrodes, as shown in the LSV profiles in Fig. 7a. Unlike the glucose oxidation, the NiFeN_*x*_-NF electrode showed a higher HER activity in 1 M KOH solution than the NiFeO_*x*_-NF and NiFe(OH)_*x*_-NF electrodes. The overpotentials for reaching current densities of 10 and 100 mA cm^−2^ by the NiFeN_*x*_-NF electrode were 40.6 and 104 mV, respectively, higher than those of 20% Pt/C catalyst (Supplementary Fig. 23). The Tafel slope of the NiFeN_*x*_-NF electrode for HER was calculated as 39 mV dec^−1^, much lower than those of the NiFe(OH)_*x*_-NF (142 mV dec^−1^) and NiFeO_*x*_-NF (97 mV dec^−1^) electrodes (Fig. 7b), further confirming that NiFeN_*x*_-NF was an efficient cathodic HER catalyst. For comparison, an in situ grown iron−nickel nitride nanostructure (Ni_3_FeN-NF) was reported to deliver 10 mA cm^−2^ of current at an overpotential of 75 mV with a Tafel slope of 98 mV dec^−1 ^^62^. The data in Supplementary Table 2 further confirm that, with the low overpotential (40.6 mV for 10 mA cm^−2^) and Tafel slope (39 mV dec^−1^) values, the NiFeN_*x*_-NF electrode is among the best performing noble-metal-free HER catalysts in alkaline solution. The EIS spectra (Supplementary Fig. 24) indicate a much lower charge-transfer resistance of the NiFeN_*x*_-NF electrode than those of the NiFeO_*x*_-NF and NF electrodes.

In order to integrate the anodic glucose oxidation with cathodic HER, the cathodic electrode must have stable catalytic activity for HER in the presence of glucose, as the glucose in the anode compartment of the electrolyzer can cross over the anion-exchange membrane into the cathode compartment. Thus, the glucose tolerance of the NiFeN_*x*_-NF electrode for HER was evaluated with the same glucose concentration found in the anode compartment. Figure 7c shows that the LSV profiles of the NiFeN_*x*_-NF electrode with and without 100 mM of glucose were very similar—only about 20 mV higher overpotential was required to reach the same current density when 100 mM of glucose was in the cathode compartment. A 24-h chronoamperometry test of HER was also conducted in the presence of 100 mM glucose at a potential of −0.135 V (Fig. 7d and right inset), which shows that the electrocatalytic current density of the NiFeN_*x*_-NF electrode remained almost unchanged, confirming its strong glucose tolerance. The LSV profiles of the fresh and 24-h-used NiFeN_*x*_-NF electrodes exhibited no difference (left inset of Fig. 5d), demonstrating the stability of the electrode.

## Discussion

**Fig. 8:**
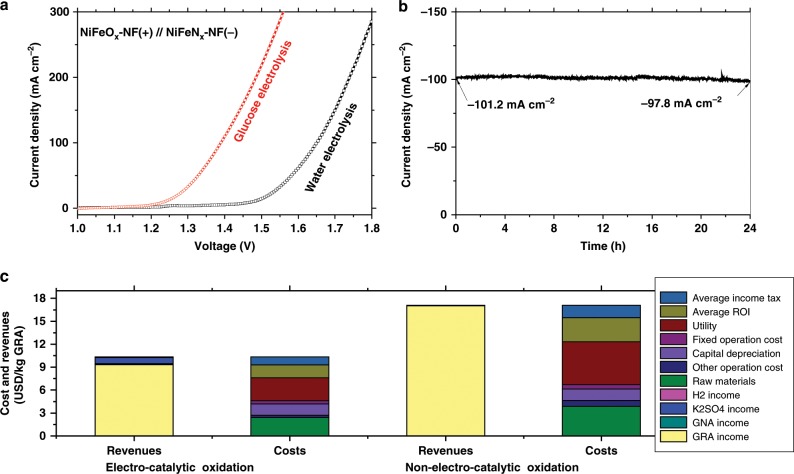
Process evaluation of the glucose electrochemical oxidation. **a** Comparison of glucose electrolysis and water electrolysis with the same anodic NiFeO_*x*_-NF and cathodic NiFeN_*x*_-NF catalysts (scan rate: 5 mV s^−1^, glucose concentration: 100 mM, 1 M KOH). **b** Long-term stability of the glucose electrolysis at a cell voltage of 1.4 V. **c** Comparison of the revenues and costs for electrocatalytic glucose oxidation and nonelectrocatalytic oxidation processes (HNO_3_ oxidation) for GRA production (1000 tons GRA per year).

A glucose electrolyzer was constructed using NiFeO_*x*_-NF as the anodic electrode and NiFeN_*x*_-NF as the cathodic electrode, separated by a anion-exchange membrane, in an H-cell with 1 M KOH and 0.5 M glucose + 1 M KOH as the anodic and cathodic electrolytes, respectively (Supplementary Fig. 25). A water electrolyzer with the same configuration was also constructed in the absence of glucose. The LSV profiles (Fig. 8a) show that the glucose oxidation electrolysis exhibited a higher current density than the water electrolysis with the same cell potential. Such low cell voltages required for glucose electrolyzer also compare favorably to the values reported for electrolyzers for the oxidation of many other organic compounds (e.g., urea, ethanol, benzyl alcohol, etc.) (Supplementary Table 3), demonstrating the higher energy efficiency of glucose electrolyzers using NiFeO_*x*_-NF and NiFeN_*x*_-NF electrodes. After 24-h at cell voltage of 1.4 V, 21.3% of the glucose was converted with the GRA and GNA yields or 11.6% and 4.7%, respectively. A high glucose concentration of 0.5 M was used to ensure that the current density did not fade in the entire electrolysis process, because the electrolysis current density would decrease with a low glucose concentration (less than 0.15 M) (Supplementary Fig. 11).

The H_2_ production in the glucose electrolyzer was observed by the evolution of bubbles at the cathode, and further confirmed by gas chromatography analysis. The long-term stability of the glucose electrolyzer was evaluated via chronoamperometry, which shows that the electrolyzer delivered a current density of 101.2 mA cm^−2^ at a voltage of 1.4 V and exhibited less than 4% decrease in this value (97.8 mA cm^−2^ remaining) after 24-h operation (Fig. 8b). It should be mentioned that in the chronoamperometry test, no bubbles were observed at the anode, confirming that the competing OER process did not occur in the glucose electrolysis process.

The economic feasibility for the electrocatalytic and nonelectrocatalytic glucose oxidation strategies was estimated assuming a production scale of 1000 tons GRA per year^63^. The minimum selling price (MSP) of GRA was calculated via a discounted cash flow analysis and setting the NPV (net present value) to 0 using the economic and technological assumptions and parameters shown in Supplementary Methods 3 and 4 and simulated with ASPEN Plus (Aspen Engineering V8.4, AspenTech, USA) (Supplementary Figs. 26–29 and Supplementary Tables 6−15). As shown in Supplementary Table 12, the electrochemical glucose oxidation has a lower capital costs ($10.5 vs. $19.3 million), lower raw material cost ($1.2 vs. $1.9 million yr^−1^), lower operating cost ($5.9 vs. 7.6 million yr^−1^) and higher revenues ($17.2 vs. 15.1 million yr^−1^) than the chemical glucose oxidation. The MSP for GRA for the electrocatalytic glucose oxidation approach is calculated to be $9.32 kg^−1^ (Fig. 8c). For comparison, the MSP of GRA for the nonelectrocatalytic oxidation process is $17.04 per kg.

In Supplementary Table 4, the electrocatalytic glucose oxidation process proposed in this work is compared with conventional chemical oxidation^23,24^ and microbial fermentation^19,20^ processes for GRA production to highlight the sustainability of glucose electrolysis. The electrocatalytic glucose oxidation process has several advantages over the other two processes because of its higher GRA yield, shorter reaction time and lower *E*-factor (the mass ratio of the generated waste to target products). The electrocatalytic glucose oxidation process has lower operation and downstream separation costs and a much smaller environmental impact. One main challenge for the electrocatalytic glucose oxidation process is that it will require large amounts of KOH (370 tons KOH for production of 1000 tons GRA) and the associated equipment. Fortunately, KOH is not directly discharged into the environment after the reaction, but converted into K_2_SO_4_ (650 tons per year), which can be sold as a byproduct to produce fertilizers.

In conclusion, an electrolysis method was developed to convert glucose into GRA and H_2_. The NiFeO_*x*_-NF and NiFeN_*x*_-NF electrodes derived from NiFe LDH nanosheet arrays demonstrate high yields toward anodic glucose oxidation and cathodic HER respectively. The low onset potentials (1.13 V) and Tafel slopes (19 mV dec^−1^) confirm that glucose oxidation is much more favorable than OER, leading to a high Faradaic efficiency (87%) toward GNA and GRA production. A two-electrode glucose electrolyzer constructed with NiFeO_*x*_-NF as the anode for glucose oxidation and NiFeN_*x*_-NF as the cathode for HER can deliver a current density of 200 mA cm^−2^ with a voltage of only 1.48 V, and run stably for 24 h, placing such a glucose electrolyzer among the best organic compound electrolyzers with noble-metal-free electrodes reported so far. Because of the abundant nature of these catalyst materials and the cost-effective and energy-saving production of value-added chemicals like GRA and H_2_, this organic compound electrolysis strategy is expected to be promising and sustainable for valorization of biomass feedstocks.

## Methods

### Synthesis of NiFe-OH nanosheets-based electrocatalysts

The chemicals and materials used in this work are listed in Supplementary Note 1. The NiFe hydroxides nanosheets were grown on the NF via a facile hydrothermal approach described as follows: FeCl_3_•6H_2_O (540.6 mg, 2 mmol) was dissolved in 10 mL of H_2_O, which was then transferred to a Teflon-lined stainless steel autoclave of 50 mL. A piece of NF was immersed into the Fe(III) solution and reacted under ultrasonic for 30 min. Urea (900 mg, 15 mmol) was dissolved in 10 mL H_2_O and mixed with the Fe(III) solution, and then 20 mL of ethanol was added into the solution. The autoclave was subsequently sealed and heated to 160 °C for 24 h. Afterwards, the NF was washed with H_2_O and ethanol several times to obtain the NiFe hydroxide nanosheets (denoted as NiFe(OH)_*x*_-NF). The NiFe(OH)_*x*_-NF was then heated in air or NH_3_-Ar gas mixture (20:80) at 300 °C (heating rate 2 °C per min) for 3 h to obtain Ni-Fe oxides (denoted as NiFeO_*x*_-NF) or nitrides (denoted as NiFeN_*x*_-NF), respectively.

### Electrochemical measurements

The glucose electrochemical oxidation was conducted with a CHI 760D electrochemical workstation (ChenHua Instrument Inc., China), in which the Ag/AgCl electrode was used as reference electrode, the as-synthesized catalysts on NF electrodes (NiFeO_*x*_-NF, NiFeN_*x*_-NF, and NiFe(OH)_*x*_-NF) were used directly as working electrodes, and a Pt wire was used as counter electrode (unless otherwise stated). The tested potential vs. Ag/AgCl can be converted into potential vs. RHE via the Nernst equation listed as follows:

$${{E}}_{{\mathrm{RHE}}} = {{E}}_{{\mathrm{Ag}}/{\mathrm{AgCl}}} + 0.059\,{\mathrm{pH}} + 0.197.$$

The water electrolysis experiments were conducted in an H-type electrochemical cell, in which the anode and cathode electrolyte were both 1 M KOH (100 mL), and an anion-exchange membrane (AMI-7001, Membranes International Inc., USA) was used to separate the cathode and anode compartments. The glucose electrolysis experiments were conducted in the same manner as water electrolysis except that the anode electrolyte was 1 M of KOH solution dissolved with glucose (0−100 mM). The polarization curves were collected with LSV at 5 mV s^−1^. The stability of the catalysts for glucose electrolysis was evaluated through chronoamperometry at 1.30 V in 100 mL of glucose solution (10 mM in 1 M of KOH) for five successive cycles. EIS were recorded at 1.3 V and −0.3 V for glucose electrolysis and HER, respectively, with a frequency range from 1 Hz to 100 kHz. The concentration profiles of glucose and its oxidation products in the electrolysis process were analyzed with an HPLC equipped with a refractive index detector.

## Supplementary information

Supplementary information

Supplementary Dataset

## Data Availability

All data are available from the corresponding authors upon reasonable request.
