# Supplementary material for: Efficient electrochemical production of glucaric acid and H2 via glucose electrolysis
Source: Nat Commun. 2020 Jan 14;11:265. doi: 10.1038/s41467-019-14157-3 (PMC6959317; doi:10.1038/s41467-019-14157-3)
Supplement: Supplementary file 1 — Supplementary information [file 41467_2019_14157_MOESM1_ESM.pdf]

Supporting Information for

Efficient electrochemical production of glucaric acid and H_2 via glucose electrolysis

Wu-Jun Liu[1,2], Zhuoran Xu[2], Dongting Zhao[2], Xiao-Qiang Pan[1], Hong-Chao Li[1], Xiao Hu[1], Zhi-Yong Fan[3], Wei-Kang Wang[3], Guo-Hua Zhao[3], Song Jin[4], George W. Huber[2,*], Han-Qing Yu[1,*]

[1]CAS Key Laboratory of Urban Pollutant Conversion, Department of Applied Chemistry, University of Science & Technology of China, Hefei 230026, China

[2]Department of Chemical and Biological Engineering, University of Wisconsin-Madison, Madison, Wisconsin 53706, United States

[3]School of Chemical Science and Engineering, Tongji University, Shanghai, China

[4]Department of Chemistry, University of Wisconsin-Madison, Madison, Wisconsin 53706, United States

Correspondence and requests for materials should be addressed to G.W.H. (gwhuber@wisc.edu) or to H.-Q.Y. (hqyu@ustc.edu.cn)

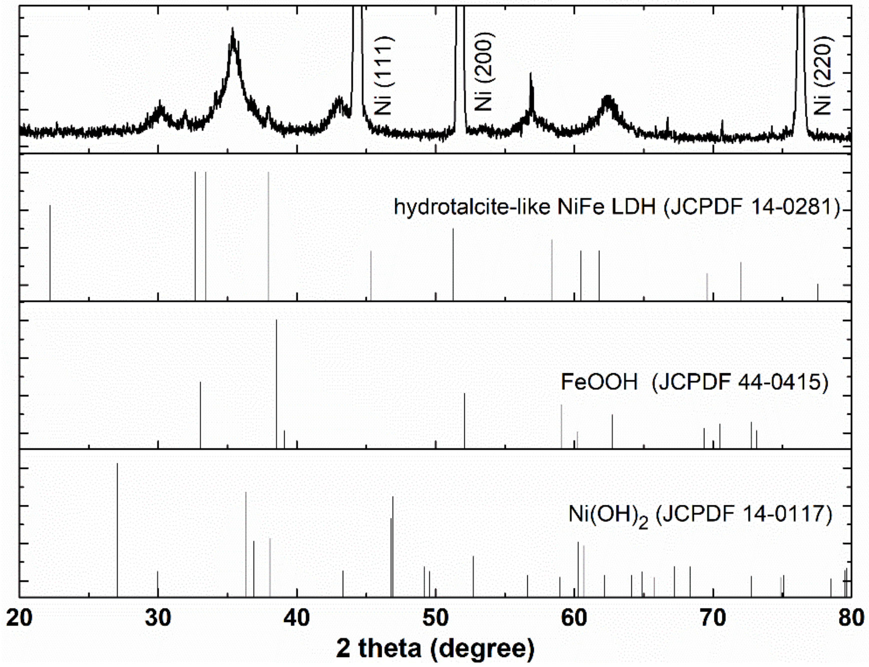

Supplementary Figure 1 | XRD pattern of the NiFe(OH)$_x$-NF.

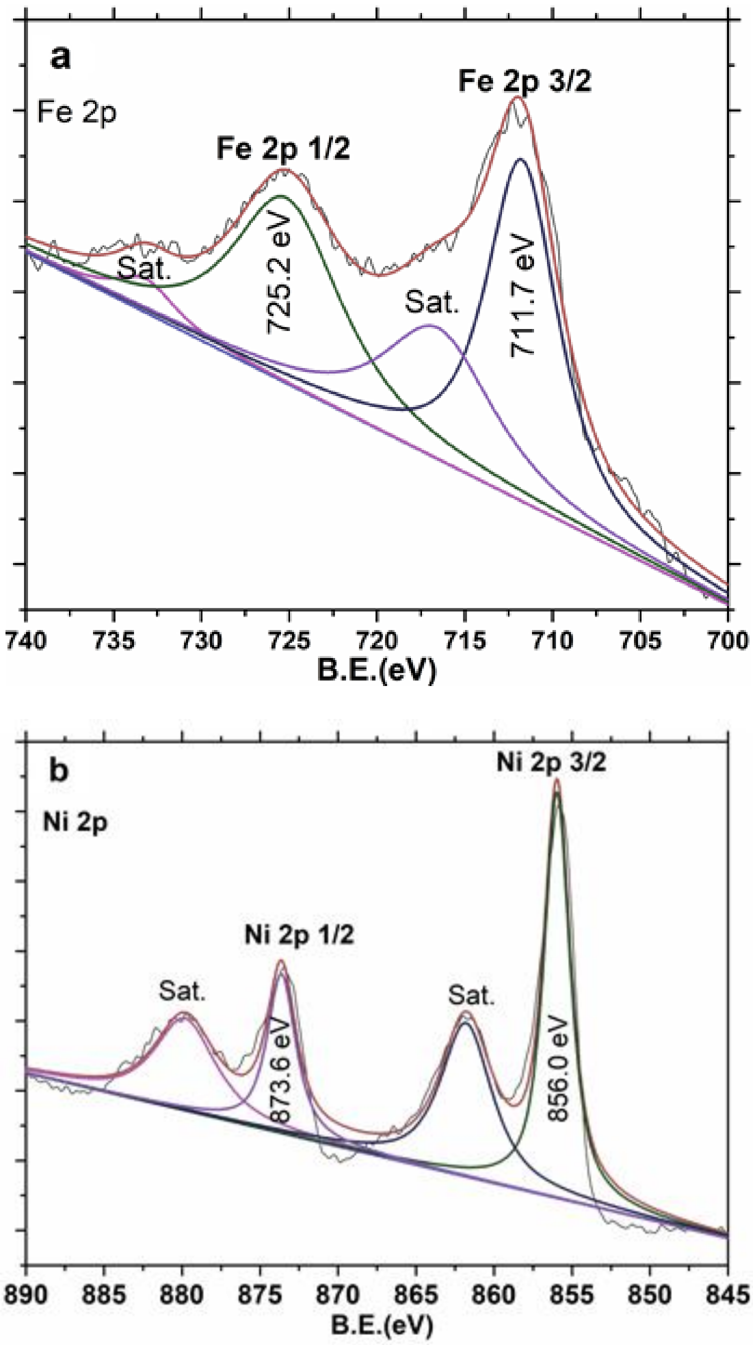

Supplementary Figure 2 | XPS analysis of the NiFe(OH)x-NF. (a) XPS Fe 2p spectrum; and (b) Ni 2p spectrum.

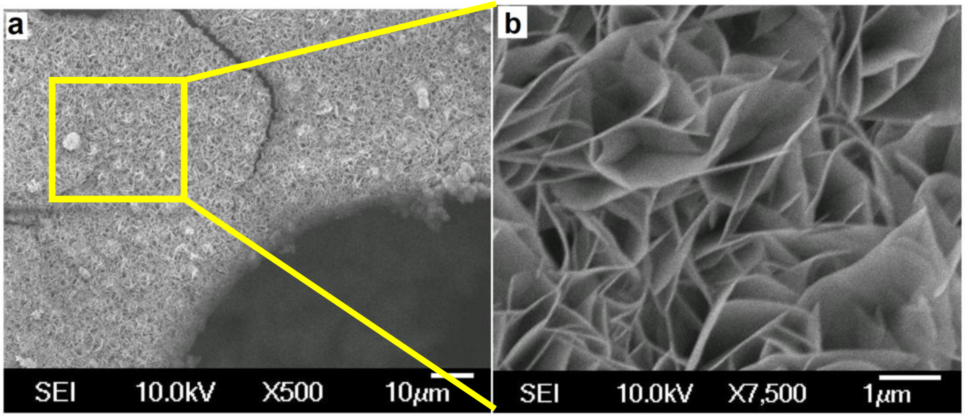

Supplementary Figure 3 | Surface morphology of the NiFe(OH)x-NF. SEM images of the NiFe(OH)$_x$-NF electrode material in low (a) and high (b) magnification.

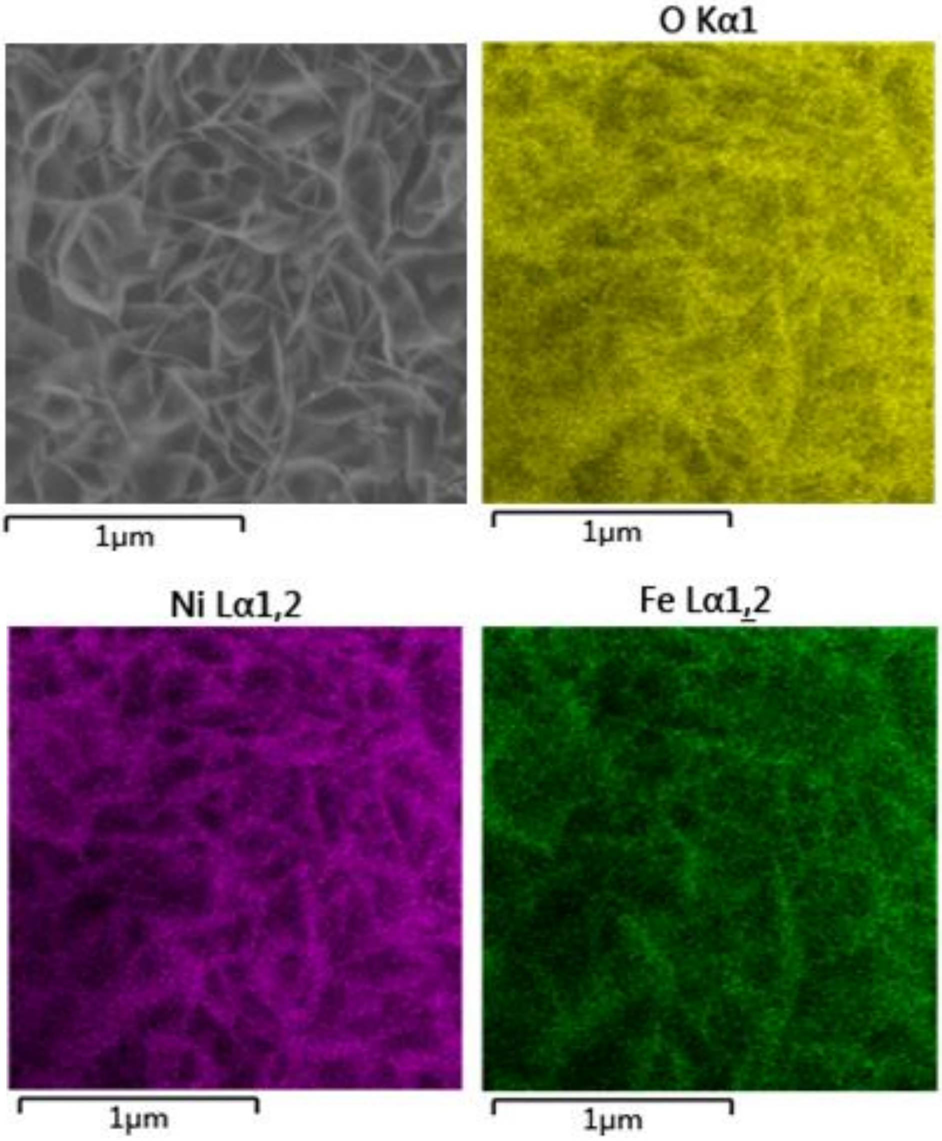

Supplementary Figure 4 | SEM-EDS elemental mapping of the $NiFe(OH)_x$-NF precursor.

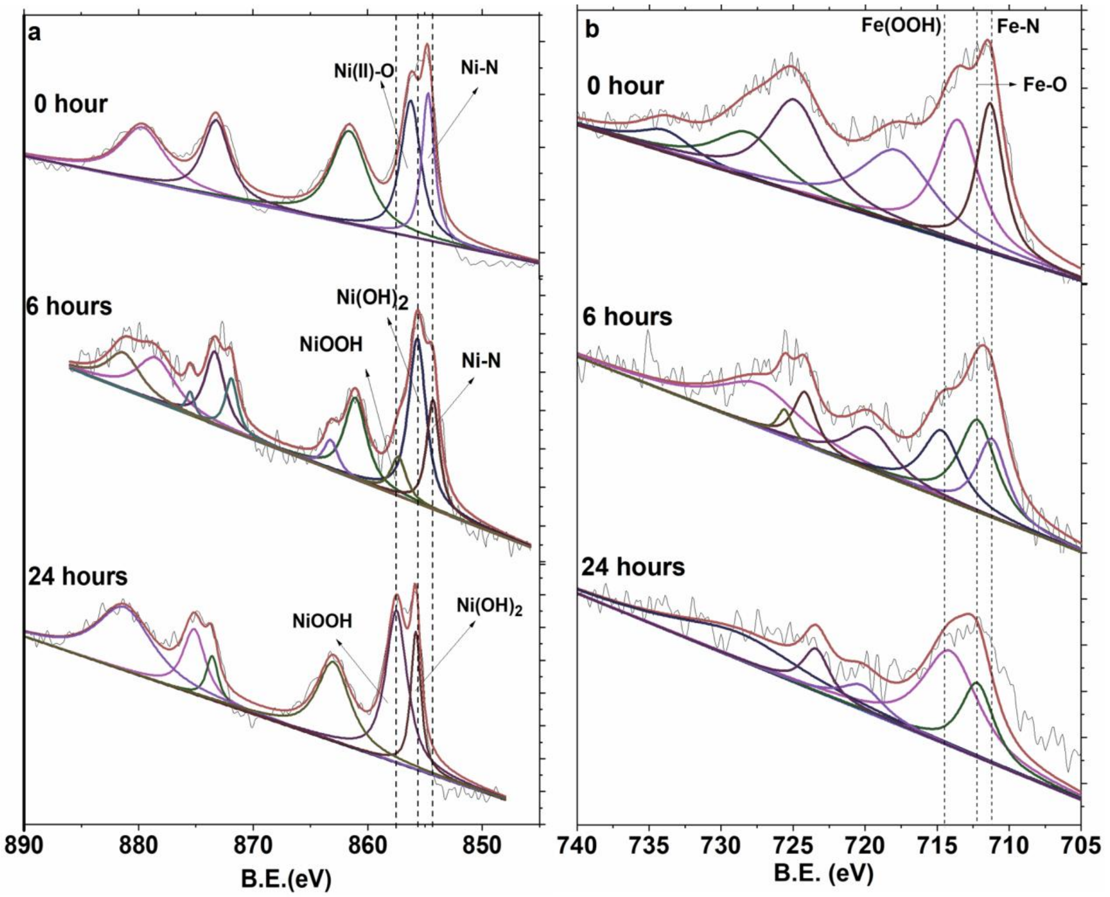

Supplementary Figure 5 | Stability of the $NiFeN_x$-NF catalyst. XPS Ni 2p (a) and Fe 2p spectra (b) of the $NiFeN_x$-NF catalyst in the electrocatalytic glucose oxidation process.

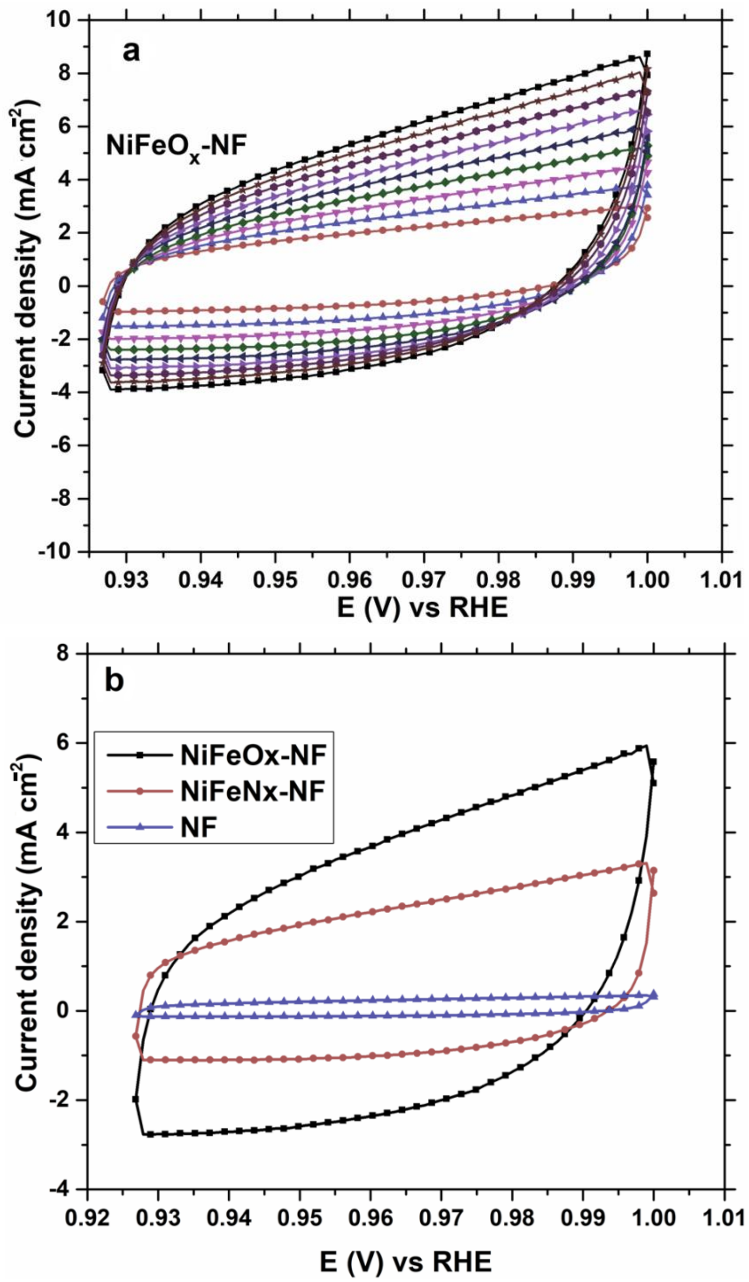

Supplementary Figure 6 | Analysis of the electrochemically active surface areas of the electrodes. (a) CV profiles of the NiFeO$_x$-NF electrode in different scan rates (10-100 mV/s from red to black symbols); and (b) CV profiles of different electrodes in the scan rate of 50 mV/s.

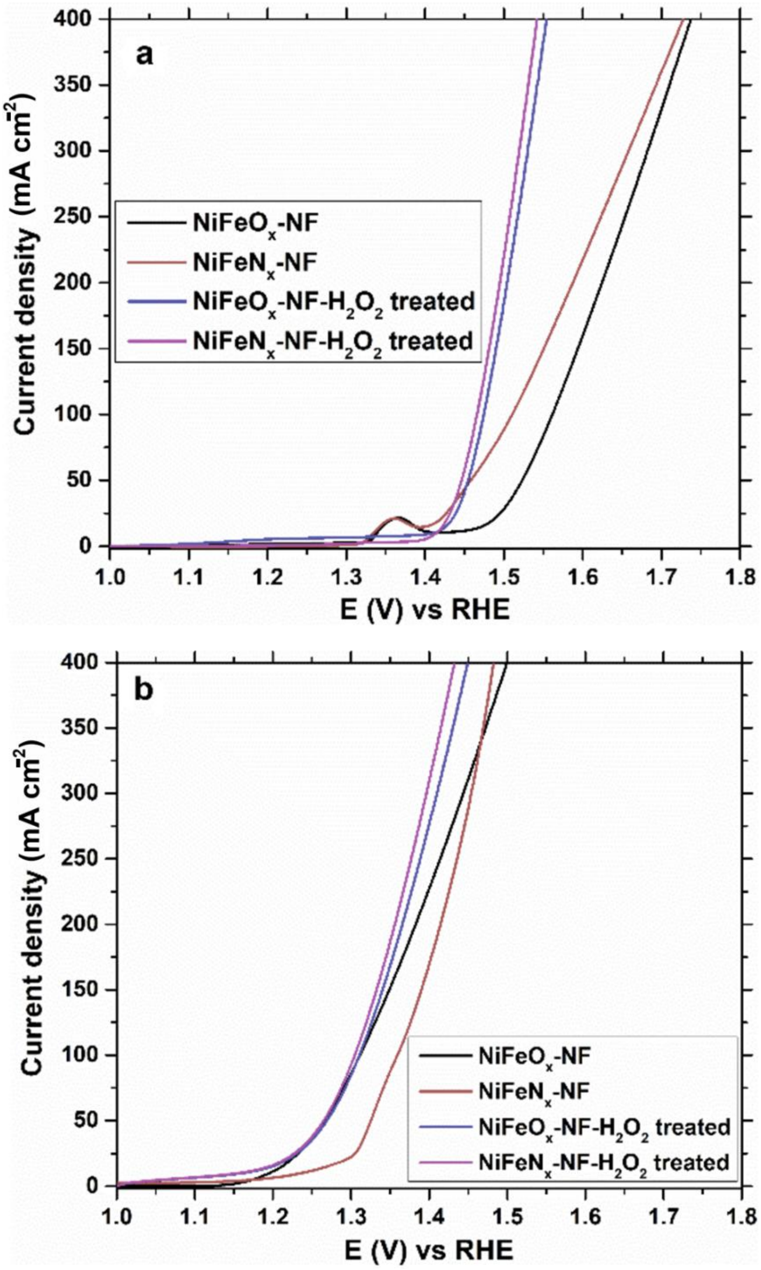

Supplementary Figure S7 | Analysis of active sites for OER and glucose oxidation. Comparison of the LSV profiles in OER (a) and glucose oxidation (b) with the catalysis of the NiFeO$_x$-NF and NiFeN$_x$-NF before and after H_2O_2 treatment

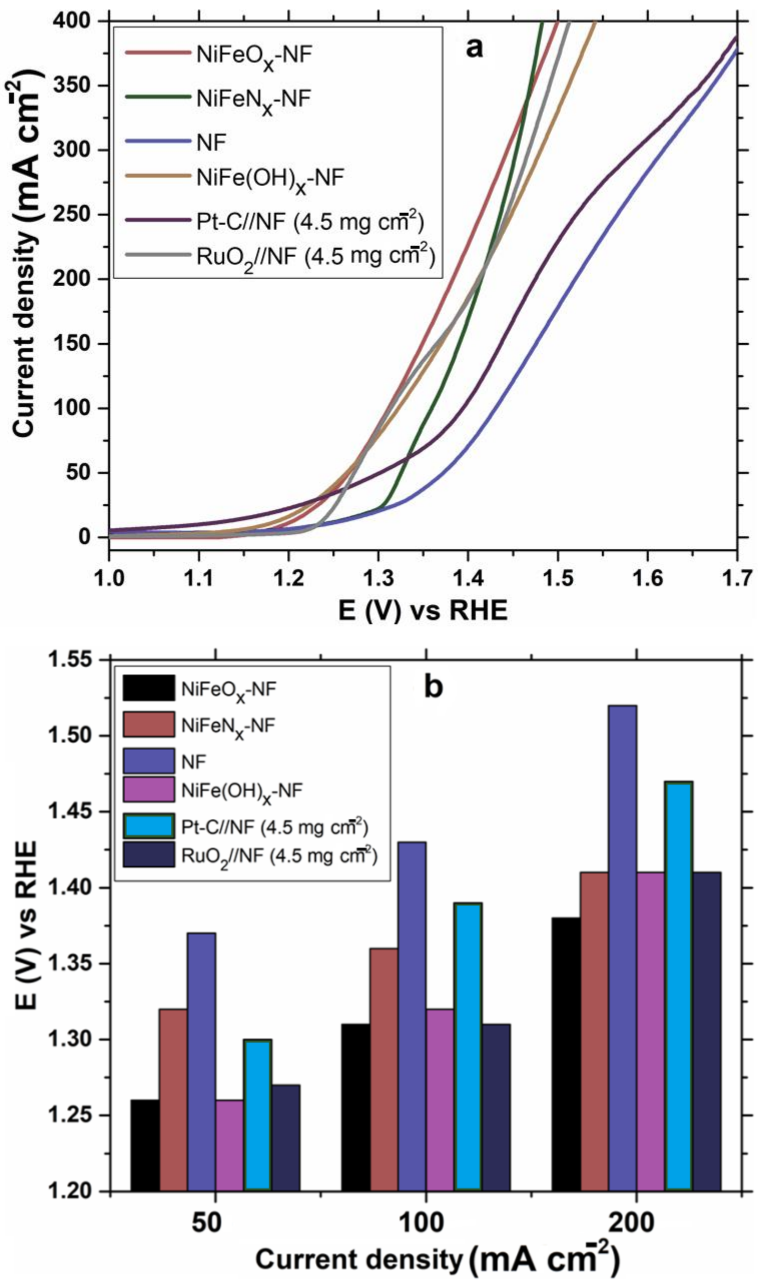

Supplementary Figure S8 | Electrocatalytic activities of different electrodes. (a) LSV profiles of the different electrode materials for glucose oxidation; (b) Comparison of the potentials in different current densities for different electrode materials.

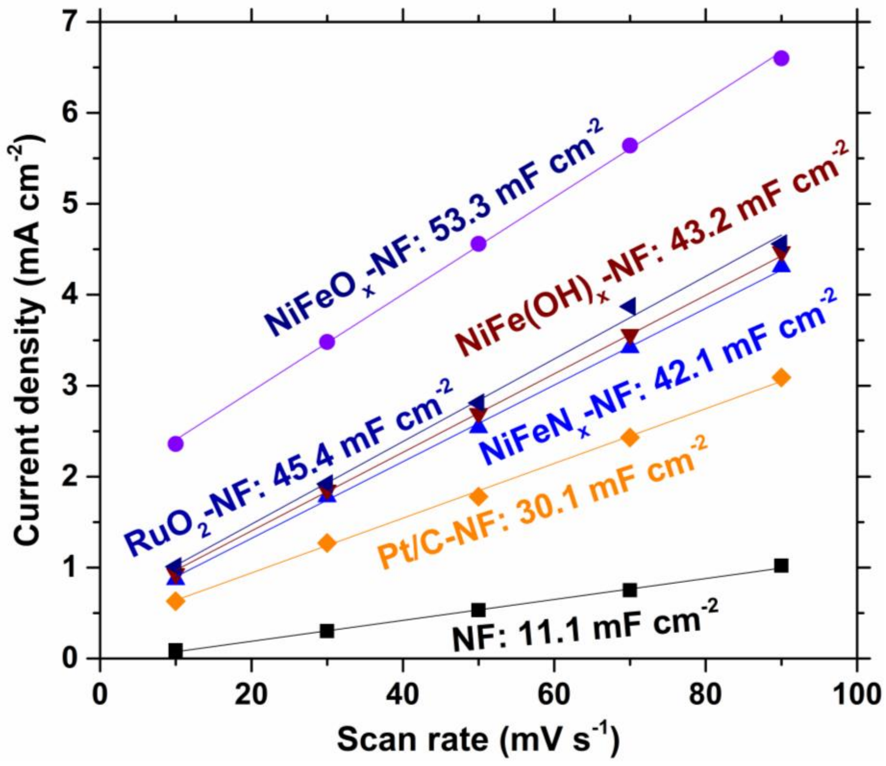

Supplementary Figure 9 | Capacitive current densities of different electrodes for glucose oxidation as a function of scan rate.

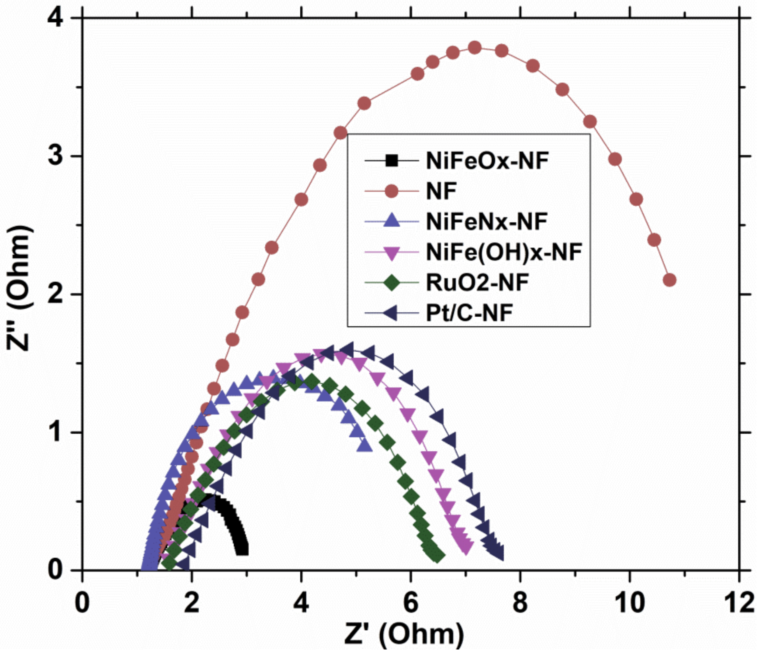

Supplementary Figure 10 | Nyquist plots (taken at 1.3 V vs. RHE) of different electrodes for glucose oxidation

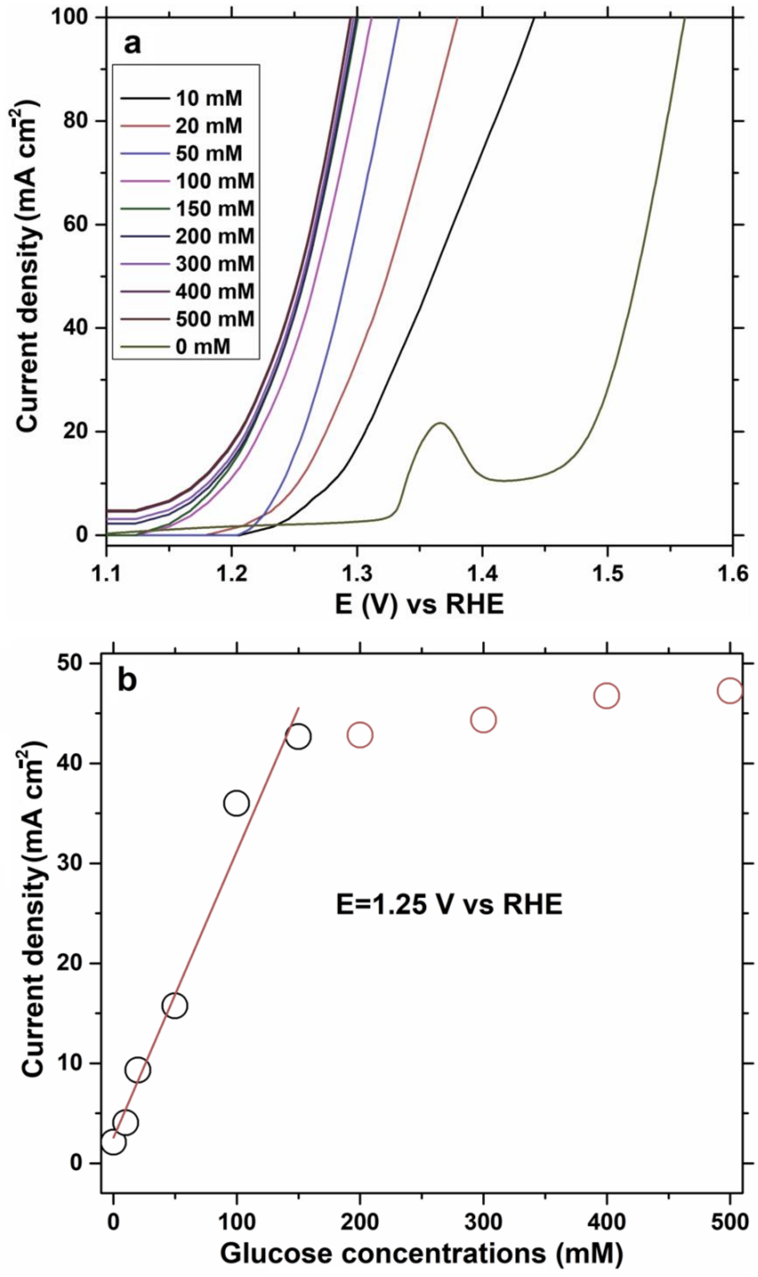

Supplementary Figure 11 | Electrochemical behaviors of the oxidation of glucose with different concentrations. (a) LSV profiles of the oxidation of glucose with different concentrations; (b) the calibration curves of the glucose concentrations with the current densities in potential of 1.25 V.

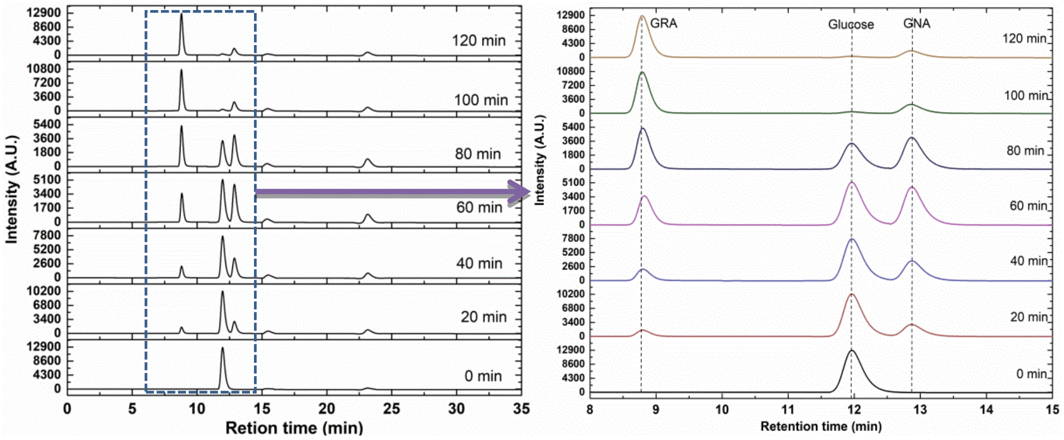

Supplementary Figure 12 | HPLC chromatograms for the glucose oxidation at different reaction times (initial glucose concentration: 10 mM)

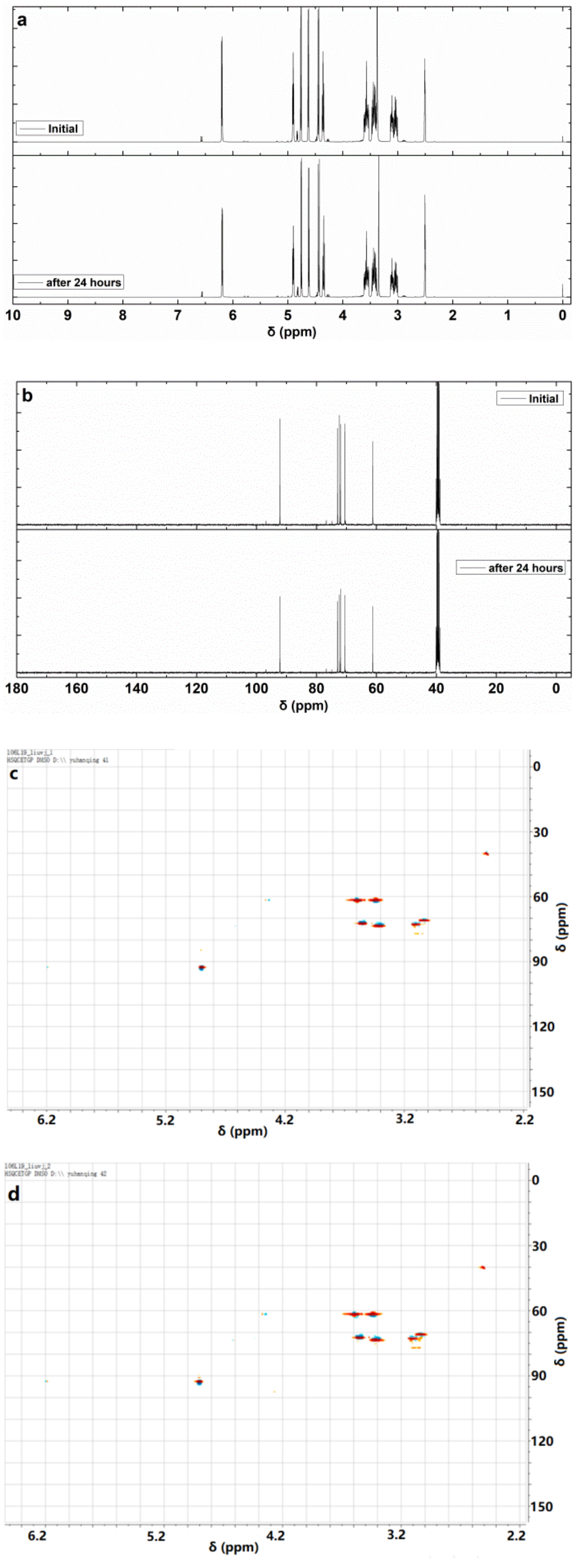

Supplementary Figure 13 | Stability evaluation of the glucose in the electrolyte. **(a)** and **(b)** ^{1}H and ^{13}C NMR spectra of glucose in its initial form and keeping after 24 h; (c) and (d) 2D-HSQC NMR spectra of the glucose in its initial form and keeping after 24 h.

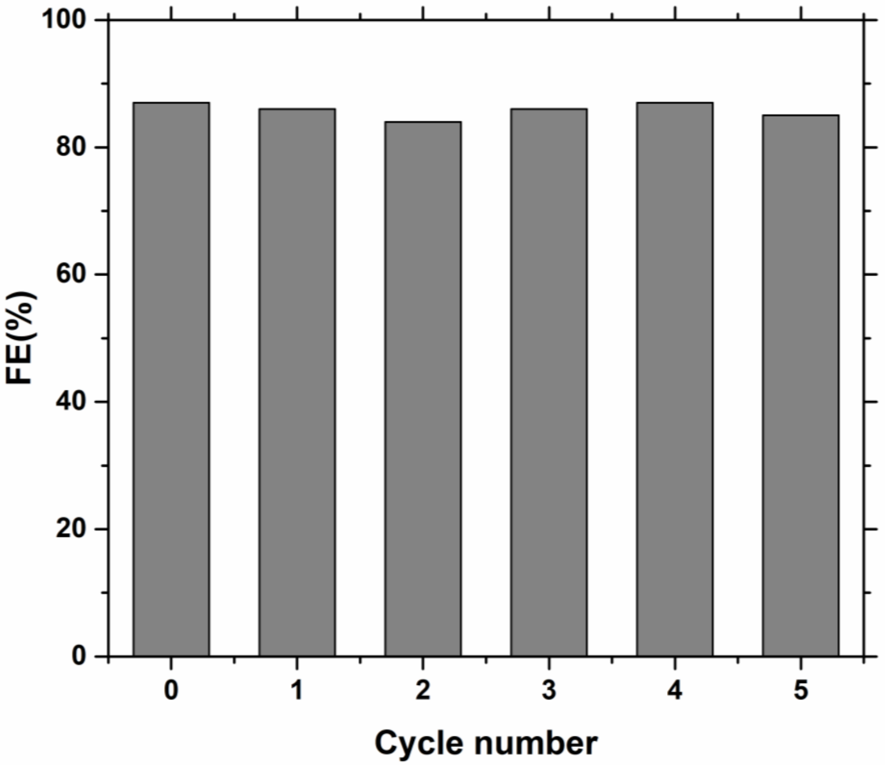

Supplementary Figure 14 | Faradaic efficiencies of the $NiFeO_x$-NF electrode in the cycle used for glucose oxidation.

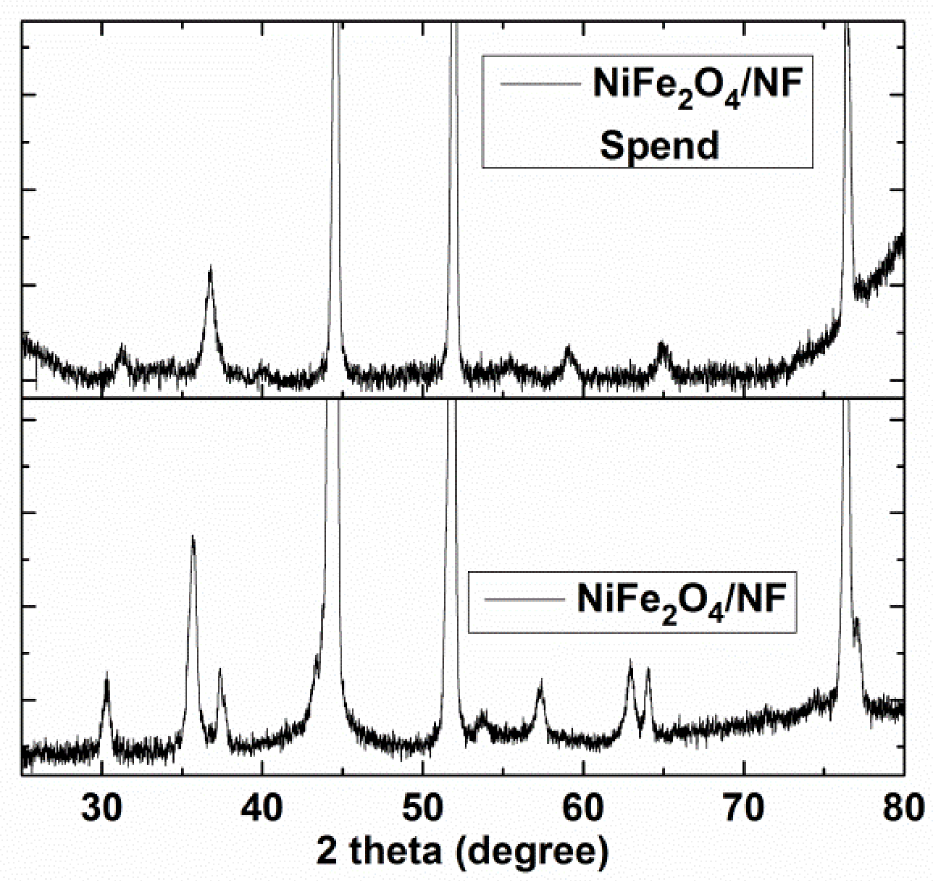

Supplementary Figure 15 | XRD patterns of the $NiFeO_x$-NF electrodes before and after cycle reused

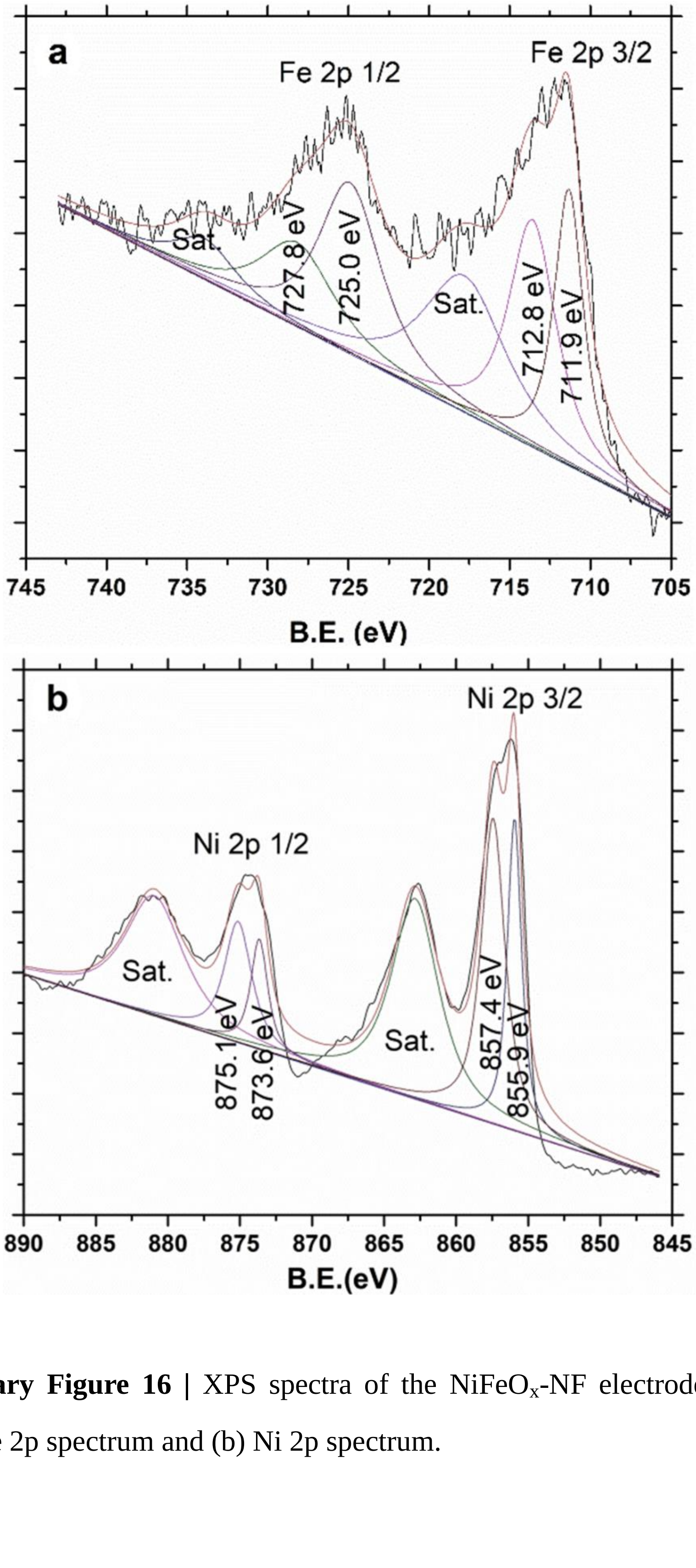

Supplementary Figure 16 | XPS spectra of the $NiFeO_x$-NF electrodes after cycle reused. (a) Fe 2p spectrum and (b) Ni 2p spectrum.

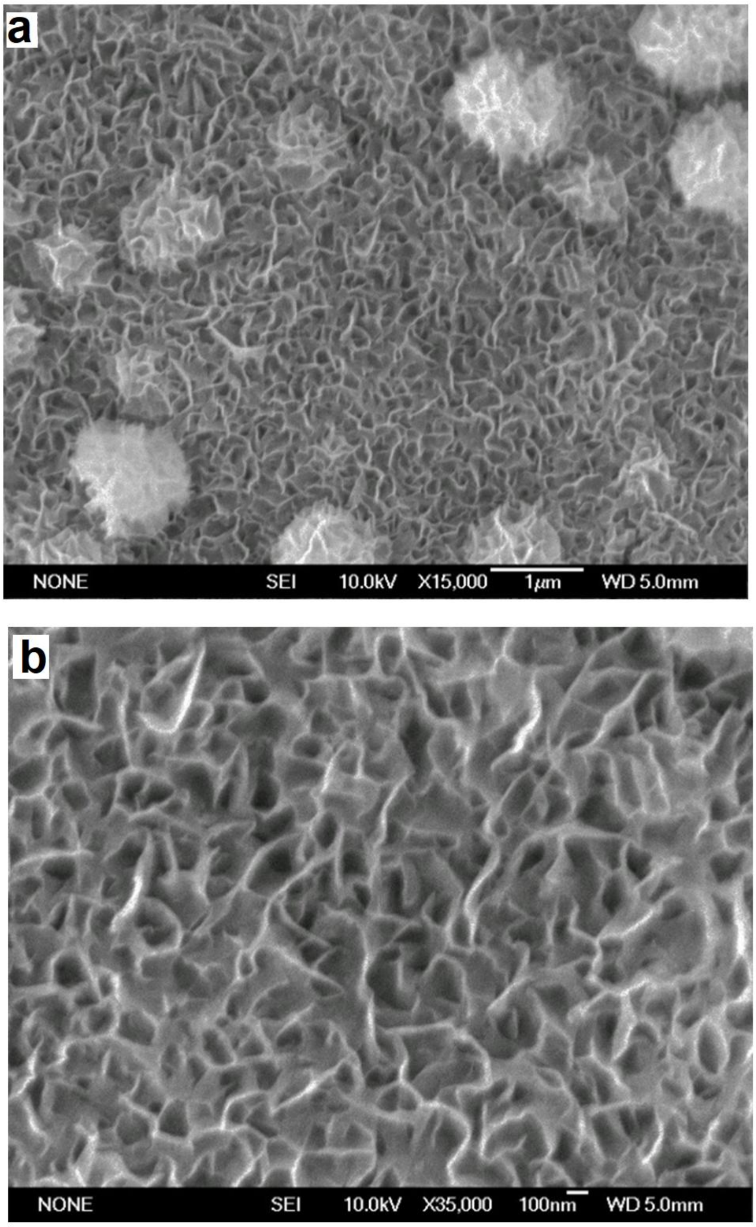

Supplementary Figure 17 | Morphology changes of the electrode after glucose oxidation. SEM image of the $NiFeO_x$-NF electrode catalyst after cycle reused in low (a) and high (b) magnification.

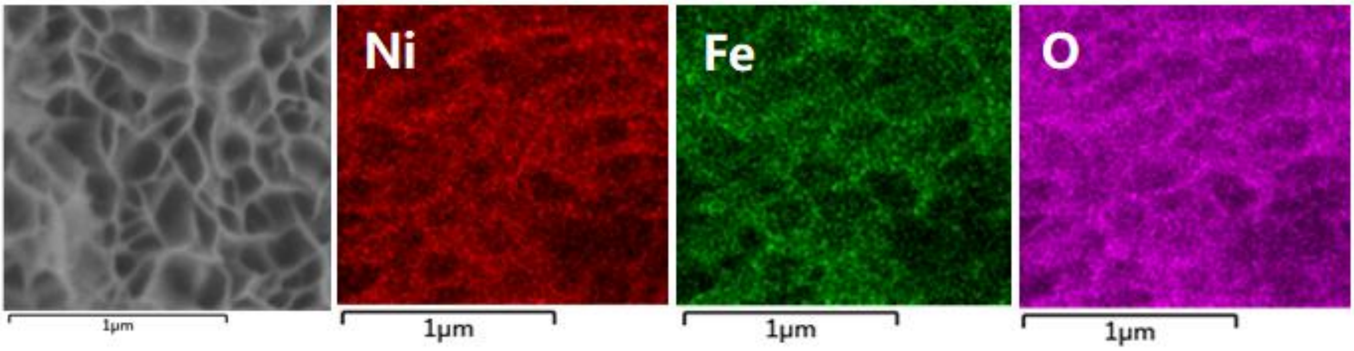

Supplementary Figure 18 | SEM-EDS elemental mapping of the $NiFeO_x$-NF electrode catalyst after cycle reused.

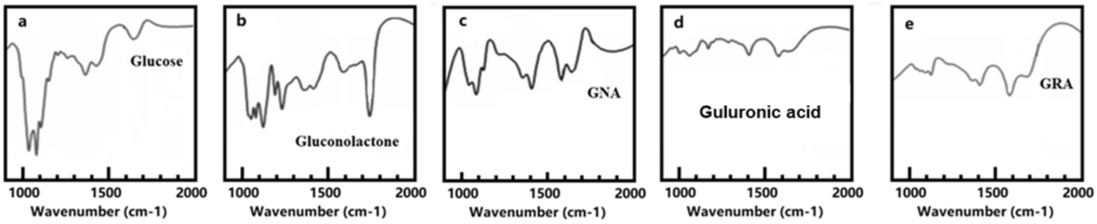

Supplementary Figure 19 | FTIR spectra of the glucose and its oxidation intermediates and products. (a) Glucose; (b) Gluconlactone; (c) GNA; (d) Guluronic acid; and (e) GRA

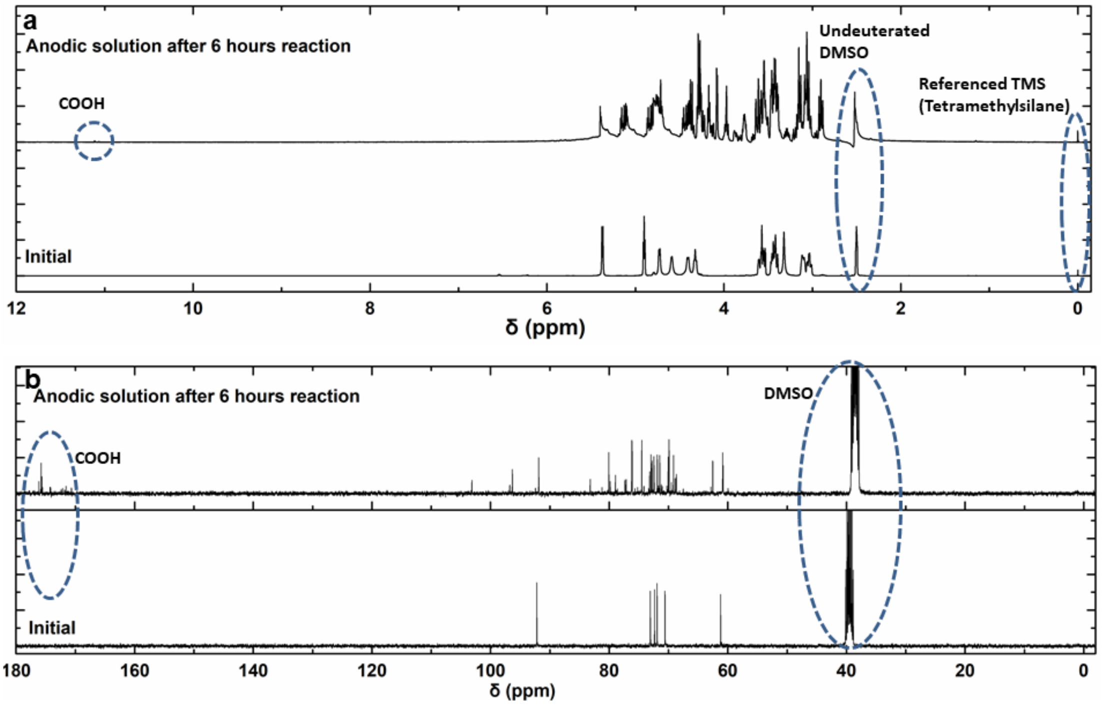

Supplementary Figure 20 | Analysis of the possible products of the glucose electrolysis process by NMR. (a) ^{1}H NMR spectra of the initial and mixtures after 6-h reaction; (b) ^{13}C NMR spectra of the initial and mixtures after 6-h reaction.

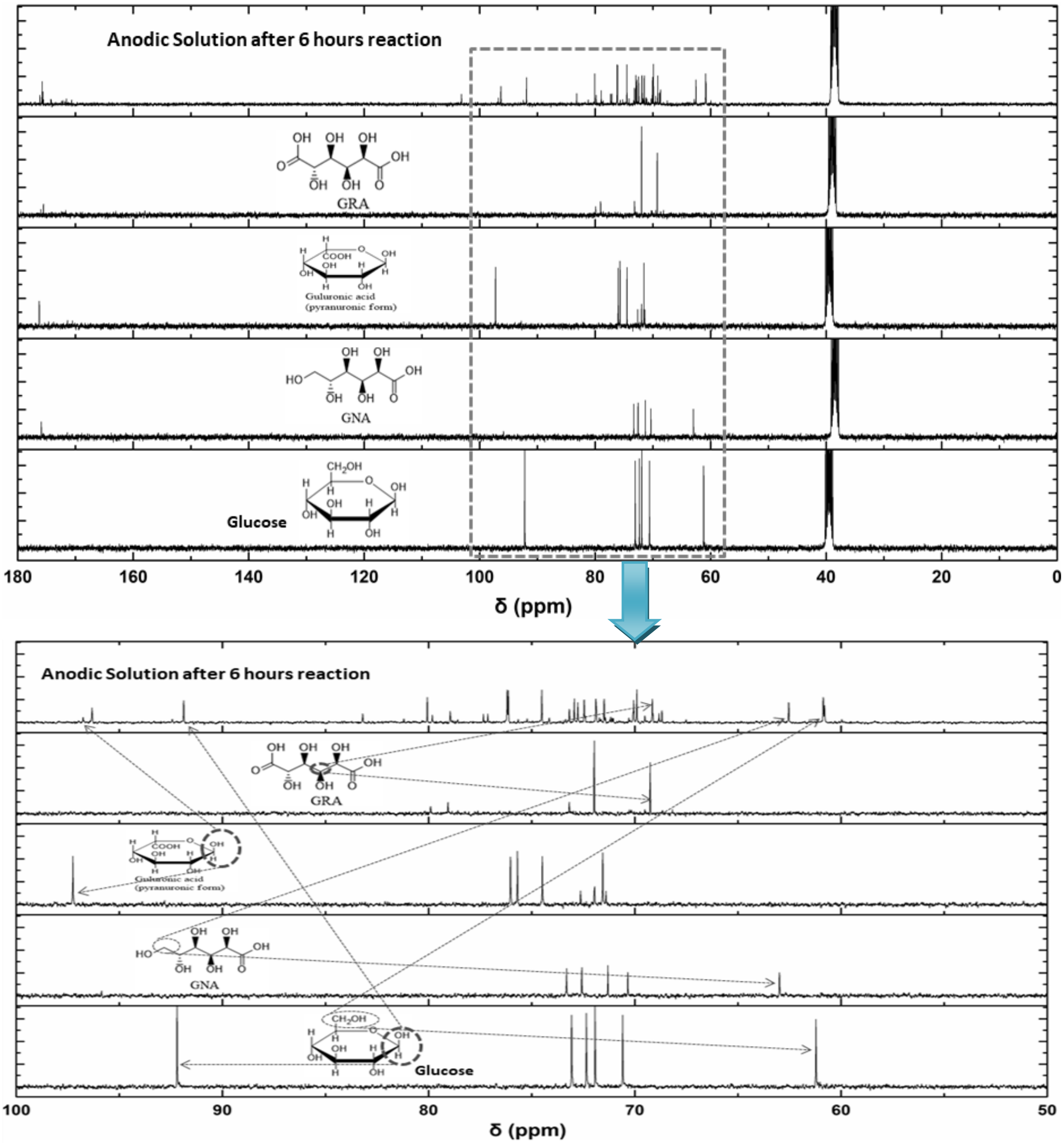

Supplementary Figure 21 | 13C NMR spectra of the standard samples of the main products of glucose oxidation (glucose, GNA, guluronic acid, and GRA) and the anodic solution after 6-h reaction.

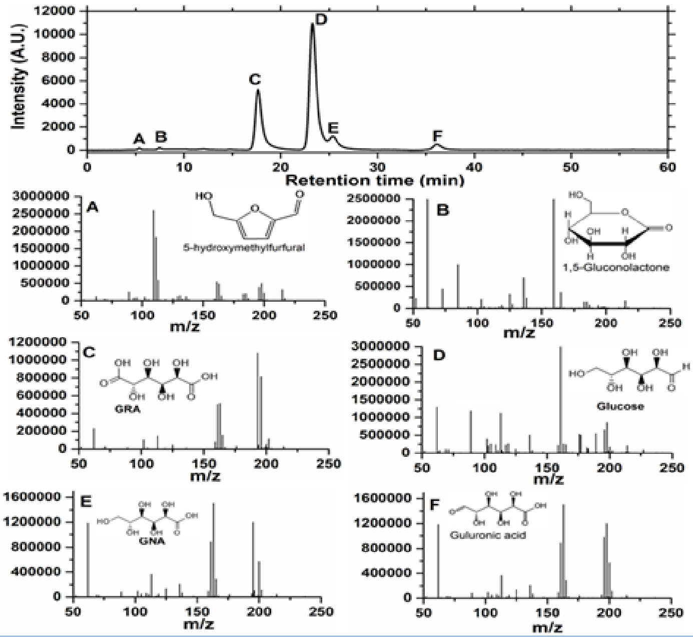

	Compounds	Formula	Molecular mass	Main ion stream
A	5-hydroxymethylfurfural	$C_6H_6O_3$	126	109 $(M+H-H_2O)^+$
B	1,5-Gluconolactone	$C_6H_{10}O_6$	178	161 $(M+H-H_2O)^+$
C	GRA (D-Glucaric acid)	$C_6H_{10}O_8$	210	193 $(M+H-H_2O)^+$<br>165 $(M+H-H_2O-CO)^+$
D	Glucose	$C_6H_{12}O_6$	180	163 $(M+H-H_2O)^+$
E	GNA (D-Gluconic acid)	$C_6H_{12}O_7$	196	197 $(M+H)^+$<br>169 $(M+H-CO)^+$
F	Guluronic acid	$C_6H_{10}O_7$	194	195 $(M+H)^+$<br>167 $(M+H-CO)^+$

Supplementary Figure 22 | LC-MS results of the reaction solution after 6-h electrolysis (initial glucose concentration: 100 mM, potential: 1.3 V).

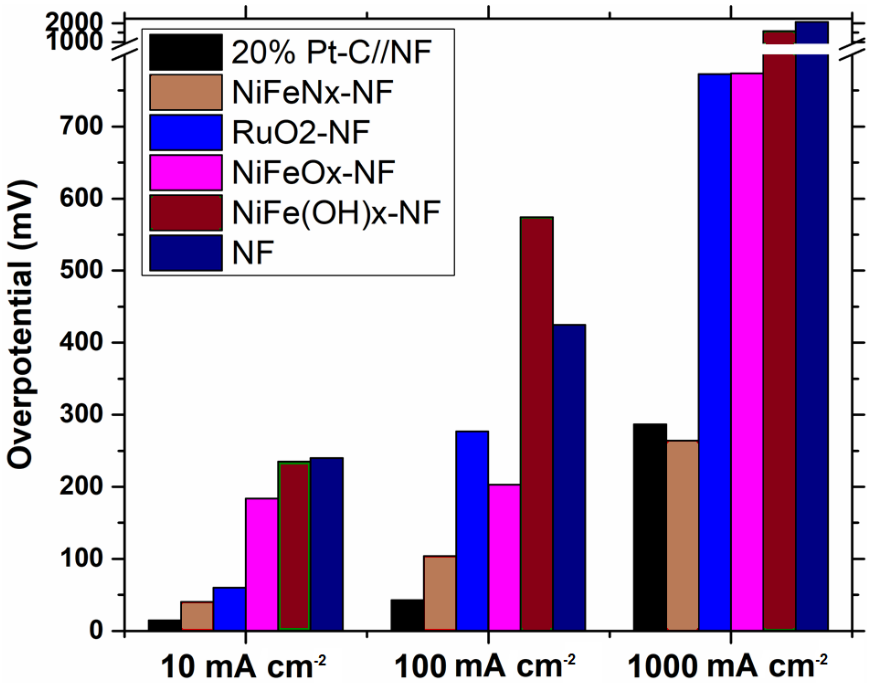

Supplementary Figure 23 | Comparison of the potentials in different current densities for different electrode materials.

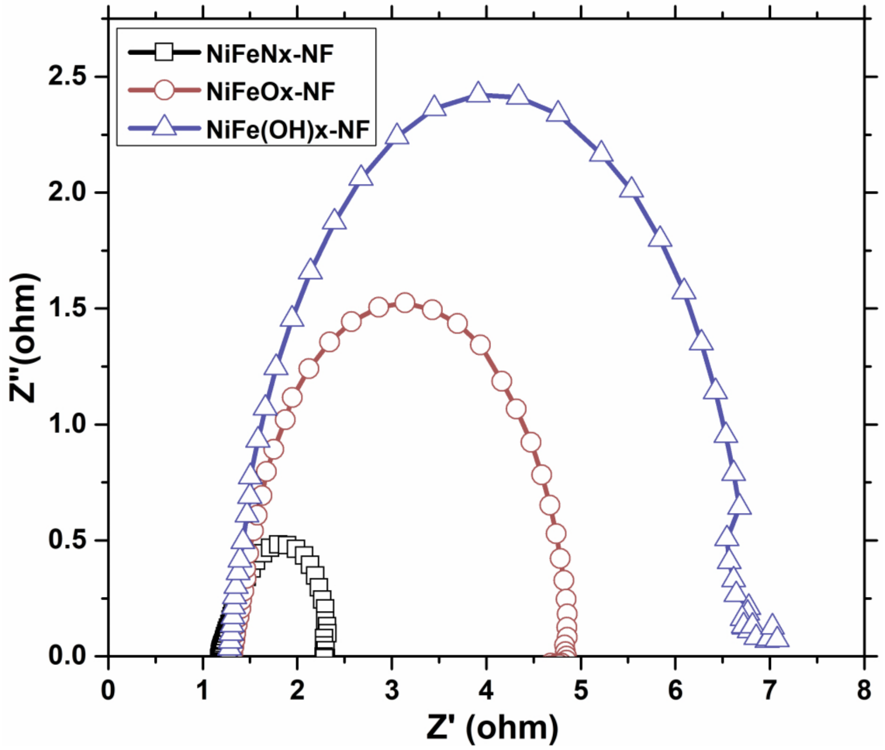

Supplementary Figure 24 | EIS spectra of the electrode catalysts under HER conditions

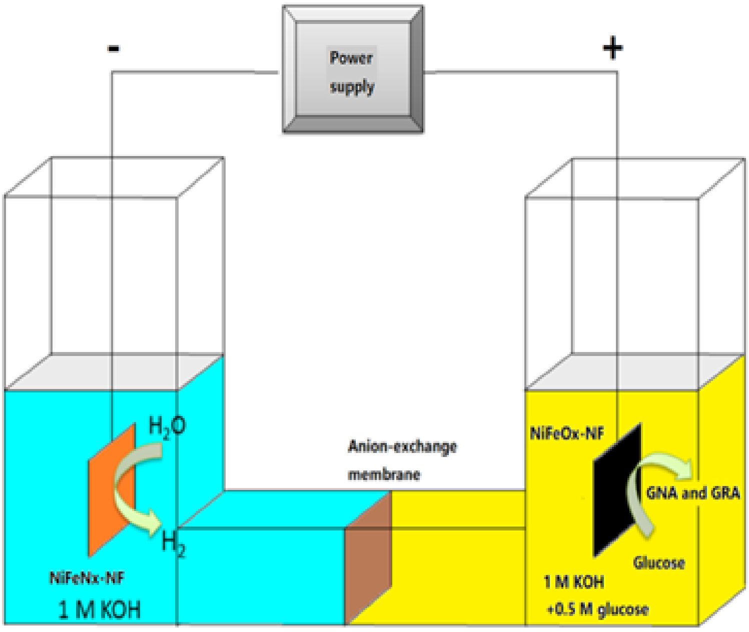

Supplementary Figure 25 | Schematic diagram of the electrochemical reactor for glucose oxidation and hydrogen production.

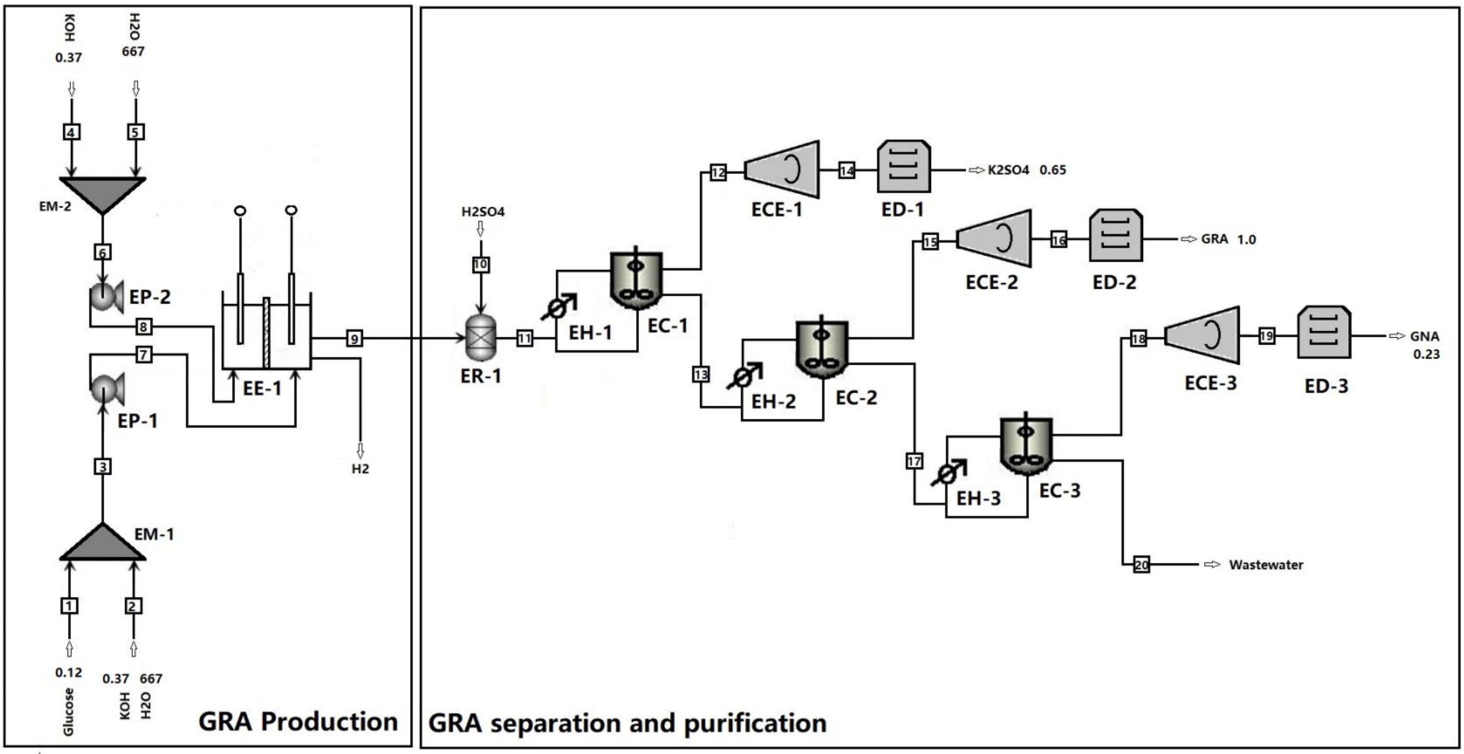

Supplementary Figure 26 | Process flow diagram of the electrocatalytic glucose oxidation process

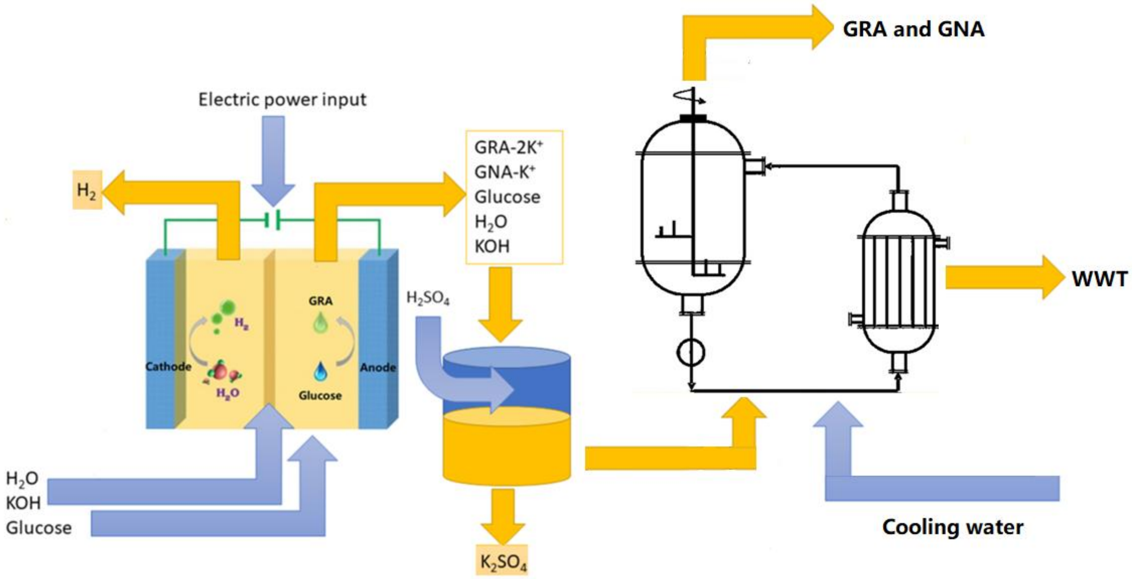

Supplementary Figure 27 | Simplified process flow diagram of the electrocatalytic glucose oxidation process

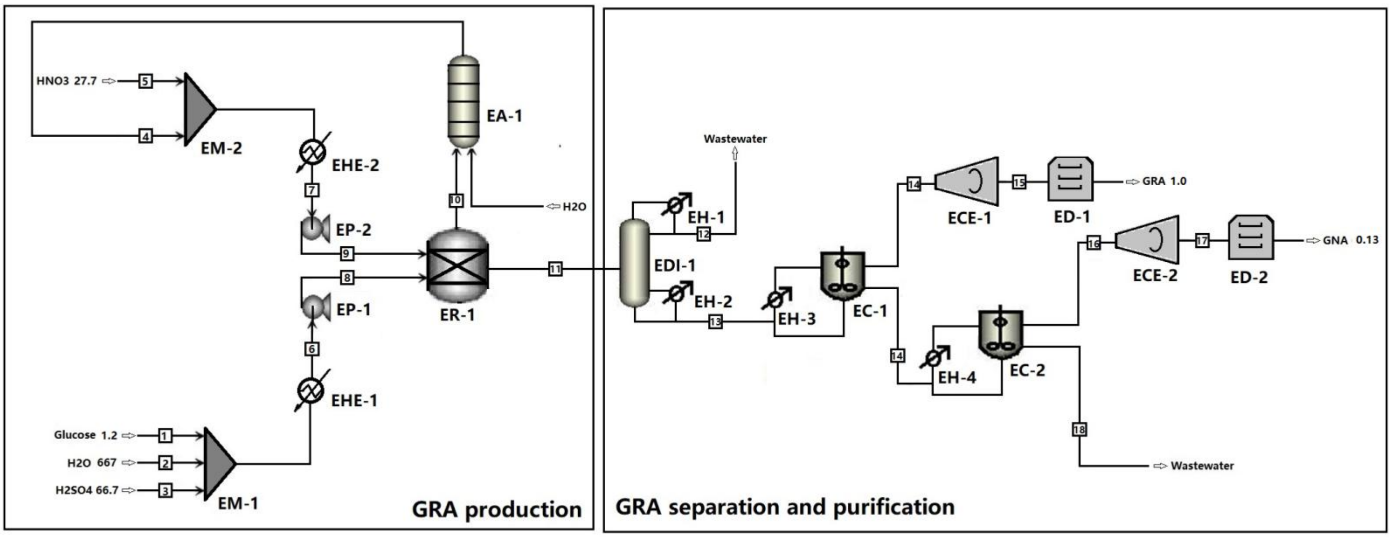

Supplementary Figure 28 | Process flow diagram of the non-electrocatalytic glucose oxidation process

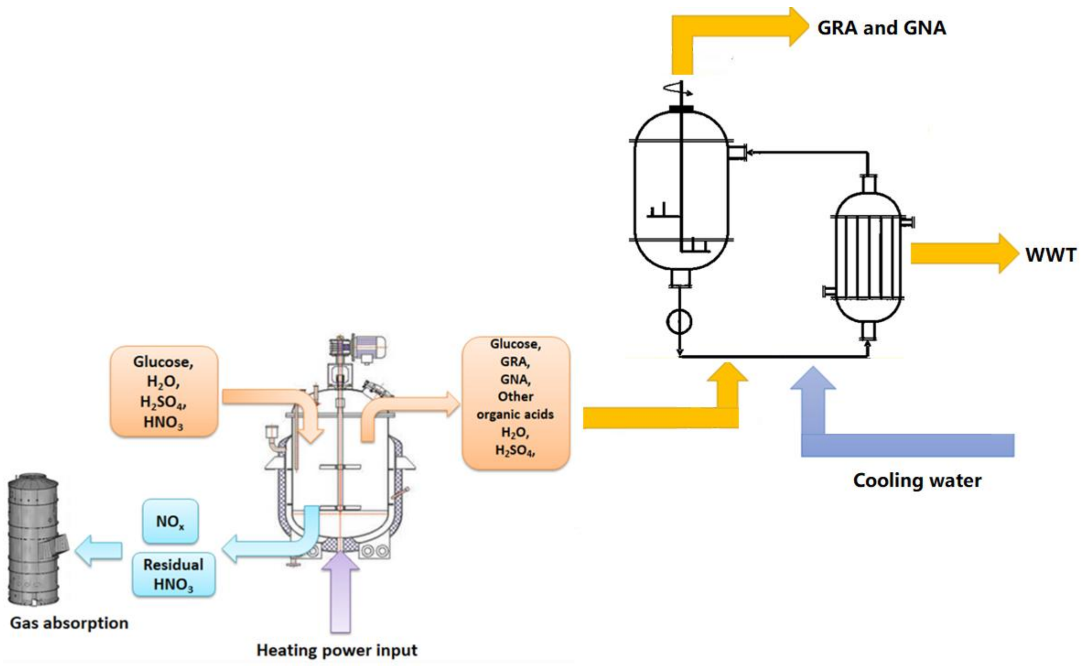

Supplementary Figure 29 | Simplified process flow diagram of the non-electrocatalytic glucose oxidation process.

Supplementary Table 1 | Band assignment of the FTIR spectra collected at different potentials in electrochemical oxidation of glucose.

Wavenumber (cm^{-1})	Assignment
1700-1800	C=O stretching in COOH
1700-1600	Characteristic absorption peak of water
1575-1506	C-O-C stretching
1483-1431	C-O-C asymmetric stretching
1397-1341	C-H bending in $-CH_2$
1283-1250	C-H bending in CH
1123	C-O stretching in CH-OH
1102-1082	C-O stretching in CH_2-OH

Supplementary Table 2 | Comparison of the $NiFeN_x$-NF catalyst with stand-of-the-art noble-metal-free catalysts for electrocatalytic HER.

Catalyst	Tafel slope (mV dec^{-1})	Current density (mA cm^{-2})	Overpotential	Electrolyte	Ref.
In-Plane 1T–2H MoS_2	65	10	250	1 M KOH	1
$Fe_{0.09}Co_{0.13}$-$NiSe_2$	89	10	92	1 M KOH	2
$Co_{1-x}Ni_xP_3$ nanoneedle arrays	87	10	60.7	1 M KOH	3
N@Mo_2C/CFP	49	10	66	1 M KOH	4
MoP@C	54	10	49	1 M KOH	5
P doped $CoSe_2$	31	10	104	1 M KOH	6
Co/Co_2P-NF	80	10	295	1 M KOH	7
	156	10	186	0.5 M H_2SO_4	
$FeNi_3N$-NF	98	10	75	1 M KOH	8
$Ni_{1-x}Fe_x$-NC	101	10	184	1 M KOH	9
CoP/Ni_5P_4/CoP-NF	58	10	71	1 M KOH	10
		100	~140		
$Ni_{11}(HPO_3)_8(OH)_6$-NF	102	10	121	1 M KOH	11
		100	274		
Ni–Co–P-NF	46	10	107	1 M KOH	12
Mesoporous FeS_2	78	10	96	1 M KOH	13
Cu NDs/Ni_3S_2 NTs-CFs	76.2	10	128	1 M KOH	14
$FeSe_2$-NF	Not mentioned	100	262	1 M KOH	15
NiMo/PVP	70	10	130	1 M KOH	16
$NiFeN_x$-NF	39	10	40.6	1 M KOH	This work
		100	104		
		1000	264		

Supplementary Table 3 | Comparison of the $NiFeN_x$-NF catalyst with stand-of-the-art noble-metal-free catalysts for electrochemical organic compound oxidation.

Catalyst	Organic compounds	products	electrolyte	Voltage (V)	Current density ($mA\ cm^{-2}$)	Ref.
Co_3O_4 nanosheets	Ethanol	Ethyl acetate	1 M KOH	1.545	50	17
F modified β-FeOOH	Ethanol	Ethyl acetate	1 M KOH	1.43	10	18
MnO_2/NF	Urea	N_2/CO_2	1 M KOH	1.40	10	19
NiMoO-Ar/NF(+) //	Urea	N_2/CO_2	1 M KOH	1.38	10	20
NiMoO-H_2/NF(-)				1.55	100	
$MnO_2/MnCo_2O_4$-NF	Urea	N_2/CO_2	1 M KOH	1.55	10	21
Ni_2P nanoflake arrays	Urea	N_2/CO_2	1 M KOH	1.35	50	22
Ni_3N nanosheet array	Urea	N_2/CO_2	1 M KOH	1.44	10	23
Co−P/CF	HMF	FDCA	1 M KOH	1.44	20	24
Ni_3S_2/NF	HMF	FDCA	1 M KOH	1.46	10	25
				1.52	20	
				1.58	50	
				1.64	100	
Ni_2P/NF	HMF	FDCA	1 M KOH	1.44	10	26
				1.58	50	
Ni_2P/Ni/NF	Furfural	2-furoic acid	1 M KOH	1.48	10	27
3D hierarchically	Benzyl	Benzoic acid	1 M KOH	1.50	10	28
porous nickel	alcohol			1.54	20	
				1.60	50	
				1.66	100	
NC@$CuCo_2N_x$/CF	Benzyl alcohol	Benzoic acid	1 M KOH	1.55	10	29
Fe_2P films	Glucose	Gluconolactone	1 M KOH	1.22	10	30
				1.40	20	
				1.51	50	
				1.58	100	
NiS_2 nanostructure film	2-propanol	acetone	1 M KOH	1.69	20	31
Co_3O_4 NWs/CC//CoP	Triclosan	Phenol,	1 M KOH	1.63	10	32
NWs/CC		1,2-dihydroxybenzene,		1.70	20	
		2-phenoxyphenol				
$NiFeO_x$-NF//$NiFeN_x$-NF	Glucose	Glucaric acid	1 M KOH	1.33	50	This
				1.39	100	work
				1.48	200	

Supplementary Table 4 | Comparison of the glucose electrolysis with chemical oxidation and microbial fermentation towards GRA production.

	Glucose electrolysis (This work)	Chemical oxidation	Microbial fermentation
Glucose concentration	0.1-0.5 mol L^{-1}	0.1-1.0 mol L^{-1}	<0.1 mol L^{-1}
GRA yield	83%	Max. 71%	<20%
Residence time	~2 hours	30 min to 3 hours	More than 2 days
Reaction medium	KOH solution	H_2SO_4 and HNO_3 solution	Water (with various nutrients for microbe growth)
Oxidant	H_2O	HNO_3, O_2, or H_2O_2	O_2
By-products	GNA	GNA, glucuronic acid, tartaric acid, acetic acid, formic acid, and hydracrylic acid	Various organic acids, soluble microbial products, and microbial biomass
Catalysts	$FeNiO_x$, $FeNiN_x$	MoO_3 or V_2O_5, noble metal catalysts	-
E-factor (kg wastes/kg product)	<0.1	1.0-5.0	25-100

Supplementary Table 5 | TOF values of the catalysts (unit: s^{-1}).

Catalyst	Glucose (100 mM) oxidation			Oxygen evolution			Hydrogen evolution		
	1.20 V	1.30 V	1.40 V	1.20 V	1.30 V	1.40 V	-0.1 V	-0.15 V	-0.2 V
NiFeOx-NF	0.02	0.16	0.41	3.1×10^{-4}	1.6×10^{-3}	0.02	3.8×10^{-6}	0.02	0.10
$NiFeN_x$-NF	0.01	0.04	0.31	3.1×10^{-3}	4.7×10^{-3}	0.03	0.13	1.50	3.01
$NiFe(OH)_x$-NF	0.02	0.09	0.20	1.7×10^{-4}	7.3×10^{-4}	9.6×10^{-3}	4.4×10^{-3}	9.2×10^{-3}	0.02
NF	1.4×10^{-4}	4.4×10^{-4}	1.7×10^{-3}	2.7×10^{-5}	5.4×10^{-5}	3.9×10^{-3}	9.9×10^{-5}	1.9×10^{-4}	3.9×10^{-4}
Pt-C//NF	0.008	0.02	0.04	1.3×10^{-5}	2.1×10^{-5}	5.9×10^{-4}	0.34	0.54	0.74
RuO_2//NF	1.7×10^{-4}	4.3×10^{-4}	9.4×10^{-3}	6.6×10^{-5}	1.4×10^{-4}	8.6×10^{-3}	3.2×10^{-3}	5.7×10^{-3}	8.8×10^{-3}

Supplementary Table 6 | Detailed mass balance of the electrocatalytic glucose oxidation process (PFD is shown in Supplementary Figure 26).

Mass/energy flow (10^3-ton/year)	Stream																			
	1	2	3	4	5	6	7	8	9	10	11	12	13	14	15	16	17	18	19	20
H_2O	0	667	667	0	667	667	667	667	667	0	667	0.65	661.5	0	10	0	651.5	0.23	0	648.2
KOH	0	0.37	0.37	0.37	0	0.37	0.37	0.37	0	0	0	0	0	0	0	0	0	0	0	0
GLU	1.2	0	1.2	0	0	0	1.2	0	0.12	0	1.2	0	1.2	0	0	0	0.12	0	0	0.12
H_2SO_4	0	0	0	0	0	0	0	0	0	3.2	0	0	0	0	0	0	0	0	0	0
K_2SO_4	0	0	0	0	0	0	0	0	0	0	6.5	0.65	0	0.65	0	0	0	0	0	0
H_2	0	0	0	0	0	0	0	0	0	0	0	0	0	0	0	0	0	0	0	0
GNA(K^+)	0	0	0	0	0	0	0	0	0.27	0	0	0	0	0	0	0	0	0	0	0
GNA	0	0	0	0	0	0	0	0	0	0	2.3	0	2.3	0	0	0	0.23	0.23	0.23	0
GRA(2 K^+)	0	0	0	0	0	0	0	0	1.33	0	0	0	0	0	0	0	0	0	0	0
GRA	0	0	0	0	0	0	0	0	0	0	10	0	10	0	10	1.0	0	0	0	0
Total mass flow	1.2	670.7	682.7	3.7	667	670.7	682.7	667.3	683	3.2	687	1.3	671.5	0.65	20	1.0	651.8	0.46	0.23	648.3
Temperature (^oC)	25	25	25	25	25	25	25	25	25	25	5	25	5	102	25	102	5	25	102	25

Supplementary Table 7 | Detailed mass balance of the non-electrocatalytic glucose oxidation process PFD is shown in Supplementary Figure 28.

Mass/energy flow	Stream																	
(10^3-ton/year)	1	2	3	4	5	6	7	8	9	10	11	12	13	14	15	16	17	18
H_2SO_4	0	0	66.7	0	0	66.7	0	66.7	0	0	66.7	0	66.7	0	0	0	0	66.7
H_2O	0	667	0	133	0	667	133	667	133	0	798	399	399	10	0	0.13	0	399
GLU	1.2	0	0	0	0	12	0	1.2	0	0	0.12	0	0.12	0	0	0	0	0.12
HNO_3	0	0	0	13.3	27.7	0	41	0	41	12.3	0	0	0	0	0	0	0	0
GNA	0	0	0	0	0	0	0	0	0	0	0.13	0	0.13	0	0	0.13	0.13	0
GRA	0	0	0	0	0	0	0	0	0	0	10	0	1.0	1.0	1.0	0	0	0
NO_x	0	0	0	0	0	0	0	0	0	4.1	0	0	0	0	0	0	0	0
Total mass flow	1.2	667	66.7	146.3	27.7	745.7	174	745.7	174	16.4	876	399	479.2	2.0	1.0	0.26	0.13	467.9
Temperature (^oC)	25	25	25	25	25	60	60	60	60	102	5	5	5	5	102	5	102	25

Supplementary Table 8 | Equipment list and installation costs for the GRA/GNA production via electrocatalytic oxidation process.

Equipment ID	Equipment name	Amount	Scale cost ($)	Installation factor	Installed cost ($)	Notes
EM	Material mixer	2	26,226	1.68	44,060	Corrosion resistance
EP	Centrifugal pump	2	125,200	2.43	304,236	Corrosion resistance
EE	Electrolyzer	20	2,104,600	1.12	2,357,152	Corrosion resistance
ER	Reactor	1	120,240	1.42	171,943	Corrosion resistance
EC	Crystallizer	3	836,190	2.58	2,157,370	-
EH	Heat exchanger	3	212,310	1.26	267,510	Cooling
ECE	Centrifugal machine	3	723,990	1.82	1,317,661	-
ED	Drying machine	3	845,100	1.48	1,250,748	-

Supplementary Table 9 | Equipment list and installation costs for the GRA/GNA production via non-electrocatalytic oxidation process.

Equipment ID	Equipment name	Amount	Scale cost ($)	Installation factor	Installed cost ($)	Notes
EM	Material mixer	2	26226	1.68	44,060	Corrosion resistance
EP	Centrifugal pump	2	125,200	2.43	304,236	Corrosion resistance
ER	Batch Reactor	20	2,508,820	1.62	4,064,288	Corrosion resistance
EHE	Heating	2	235,200	1.46	343,392	-
ED	Distillation column	1	579,020	2.38	1,379,258	-
EH	Heat exchanger	4	283,080	1.26	356,680	Cooling
ECE	Centrifugal machine	2	482,660	1.82	878,441	-
ED	Drying machine	2	563,400	1.48	833,832	-
AT	Absorption tower	1	1,562,680	2.66	4,187,982	Corrosion resistance

Supplementary Table S10 | Total direct costs, indirect cost and capital investment for a 1 kton/year process to produce glucaric acid.

	Notes	Electrocatalytic glucose oxidation ($)	Non-electrocatalytic glucose oxidation ($)
GRA/GNA production	-	2,705,448	4,755,976
GRA/GNA separation and purification	-	5,165,232	8,120,005
Products storage	-	693,290	585,320
Heat exchange	Heating and cooling	1,298,550	1,736,520
WWTs	Wastewater treatments	1,982,680	2,658,730
Others	utility	964,290	1,532,870
**Total installed cost**		**12,809,490**	**19,389,421**
Warehouse (4.0% of ISBL)	Inside-battery-limits (ISBL): cost for products production, separation and purification	314,827	515,039
Site Development (9.0% of ISBL)		708,361	1,158,838
Additional Piping (4.5% of ISBL)		354,180	579,419
**Total Direct Cost (TDC)**	Total installed cost + Warehouse + Site Development + Additional Piping	**1,4186,858**	**21,642,717**
Prorateable Expenses	10% of TDC	1,418,686	2,164,272
Field Expenses	10% of TDC	1,418,686	2,164,272
Home Office & Construction Fee	20% of TDC	2,837,372	4,328,574
Project Contingency	10% of TDC	1,418,686	2,164,272
Other Costs (Start-Up, Permits, etc.)	10% of TDC	1,418,686	2,164,272
**Total Indirect Cost (TIC)**	-	**8,512,116**	**12,985,662**
**Fixed Capital Investment (FCI)**	Including the capital cost growth (0.64)	**35,467,146**	**54,106,842**
Land	-	432,580	432,580
Working Capital	5% of FCI	1,773,357	2,705,342
**Total Capital Investment (TCI)**	**FCI + Land + Working capital**	**37,673,083**	**57,244,342**

Supplementary Table S11 | List of the economic parameters and assumptions for a 1.0 kton/year process to produce glucaric acid.

Economic parameters	Values
Glucose price ($ per ton) [a]	465
GNA price ($ per ton) [b]	550
H_2SO_4 price ($ per ton) [d]	45
HNO_3 price ($ per ton) [e]	318
KOH price ($ per ton) [f]	1280
H_2 price ($ per ton) [g]	2790
K_2SO_4 price ($ per ton) [h]	473
Process water price ($ per ton) [i]	0.22
Electricity price ($ per kWh) [j]	0.07
Low pressure steam price ($ per kWh) [k]	0.021
Cooling water price ($ per kWh) [l]	0.003
Economic assumptions	
Cost year basis [m]	USD-2015
Operating hours (hours per year) [n]	8400
Equipment life span (years) [n]	30
Working capital (% of fixed capital investment) [n]	10
Equity (% of fixed capital investment) [n]	40
Loan interest (%) [n]	8
Loan terms (years) [n]	10
Internal Rate of Return (%) [n]	10
Tax rate (%) [n]	35
Depreciation period (years) [n]	7
Replacement interval (years) [o]	7
Specified yearly replacement cost (% of installed cost of electrocatalytic reactor) [o]	15
Unplanned replacement cost (% of FCI of electrocatalytic reactor) [o]	0.5

[a] Taken from online market data (http://jiage.molbase.cn/hangqing/37063)

[b] Taken from online market data (http://jiage.molbase.cn/zuixin/?cas_num= %E8%91%A1%E8%90%84%E7%B3%96%E9%85%B8%E9%92%A0)

[d] Taken from online market data (http://jiage.molbase.cn/hangqing/25732)

[e] Taken from online market data (http://jiage.molbase.cn/hangqing/4344)

[f] Taken from online market data (http://jiage.molbase.cn/hangqing/4426)

[g] Taken from a study by the EERE's (EERE fuel cell technologies office multi-year research, development, and demonstration plan; U.S. Department of Energy: Washington, US, 2015)

[h] Taken from online market data (http://jiage.molbase.cn/hangqing/610272)

i Government set industrial water price (http://tazlh.zjzwfw.gov.cn/art/2014/6/16/art_30833_28099.html)

[j] Government set electricity price (http://www.ndrc.gov.cn/gzdt/201905/t20190515_936212.html)

[k] Taken from a book by Seider et al. (Product and process design principles : synthesis, analysis, and evaluation. 2nd ed.; Wiley: New York, United States of America, 2004; p xviii, 802 p)

[l] Government set industrial water price (http://tazlh.zjzwfw.gov.cn/art/2014/6/16/art_30833_28099.html)

[m] All costs are updated to 2015 cost levels using the Chemical Engineering Plant Cost Index (CEPCI) and the average Producer Price Index (PPI)

[n] Taken from a study by Humbird et al. (Process design and economics for biochemical conversion of lignocellulosic biomass to ethanol: dilute-acid pretreatment and enzymatic hydrolysis of corn stover;S44 National Renewable Energy Laboratory (NREL): Colorado, US, 2011)

[o] Capital investment is spread over 3 years at a rate of 8%, 60%, and 32% in the first, second, and third years, respectively.

Supplementary Table 12 | Operating costs, and revenue streams of the electrocatalytic and non-electrocatalytic glucose oxidation processes for a 1 kton/year process to produce glucaric acid.

	Notes	Electrocatalytic glucose oxidation ($ year^{-1})	Non-electrocatalytic glucose oxidation ($ year^{-1})
Glucose	-	5,580,000	7,160,100
KOH	-	4,730,600	-
H_2SO_4	-	140,400	3,001,500
HNO_3	-	-	7,218,600
Process water	-	301,840	350,570
Wastewater treatments chemicals	-	1,312,300	1,580,500
**Sum Raw materials costs**		**12,065,140**	**19,311,270**
Steam	-	1,789,200	5,425,900
Cooling water		998,200	1,569,800
Electricity	-	9,724,600	512,100
**Sum Utilities**	Steam + Cooling water + Electricity	**12,512,000**	**7,507,800**
Fixed operating costs	-	1,995,830	2,571,650
Other operating costs	-	137,200	356,890
**Total Operating costs**	Raw materials costs + Utilities + Fixed operating costs + Other operating costs	**26,710,170**	**29,747,610**
GNA sale	-	1,276,000	616,000
K_2SO_4 sale		8,320,000	-
H_2 sale		860,490	-
**Revenues (R)**	-	**10,456,490**	**616,000**

Supplementary Table 13 | Template for discounted cash flow calculations.

Year (0 = SU)	Investment or salvage value	Working capital	Start-up capital	Net manufacturing cost	Total cash flow	Discount factor
**-2**	$-A_{-2}FC$				$-A_{-2}FC$	$(1+i)^{2}$
**-1**	$-A_{-1}FC$				$-A_{-1}FC$	$(1+i)^{1}$
**0**	$-A_{0}FC$	-WC	-SU		$-A_{-0}FC$-WC-SU	$(1+i)^{0}$
**1**				TR_c (R1−C1) + TR D1	$B_1[TR_c$ (R1−C1)] + TR D1	$(1+i)^{-1}$
**2**				TR_c (R2−C2) + TR D2	$B_2[TR_c$ (R2−C2)] + TR D2	$(1+i)^{-2}$
**3**				TR_c (R3−C3) + TR D3	$B_3[TR_c$ (R3−C3)] + TR D3	$(1+i)^{-3}$
**4**				TR_c (R4−C4) + TR D4	$B_4[TR_c$ (R4−C4)] + TR D4	$(1+i)^{-4}$
**5**				TR_c (R5−C5) + TR D5	$B_5[TR_c$ (R5−C5)] + TR D5	$(1+i)^{-5}$
**6**				TR_c (R6−C6) + TR D6	$B_6[TR_c$ (R6−C6)] + TR D6	$(1+i)^{-6}$
**7**				TR_c (R7−C7) + TR D7	$B_7[TR_c$ (R7−C7)] + TR D7	$(1+i)^{-7}$
**8**				TR_c (R8−C8) + TR D8	$B_8[TR_c$ (R8−C8)] + TR D8	$(1+i)^{-8}$
**9**				TR_c (R9−C9) + TR D9	$B_9[TR_c$ (R9−C9)]+ TR D9	$(1+i)^{-9}$
**10**	SV	WC		TR_c (R10−C10) + TR D10	$B_{10}[TR_c$ (R10−C10)] + TR D10 + SV + WC	$(1+i)^{-10}$

FC = Fixed Capital;

A_{-j} = Fraction allocation of FC in Year –j; A_{-2}=8%, A_{-1}=60%, and A_0=32%;

WC =Working Capital;

SU = Start-Up Capital;

SV = Salvage Value;

R_j = Revenues in Year j;

C_j = Operation Costs in Year j;

TR = Tax Rate; TR_c = (1 − TR) = Complement of Tax Rate;

B_{+j} = Fraction of profits received in Year j; B1=0.45, B2=0.65, B3=0.85, B4 = B5 = ... B10 = 1;

D_j = Depreciation in Year j; D = D1 = D2 = ... = D10 = 0.1(FC + SU) for straight-line depreciation and a 10-year lifetime;

ER = Enterprise Rate;

CR = Construction Loan Rate;

FR = Finance (Bond) Rate

Supplementary Table 14 | Discounted cash flow for the electrocatalytic glucose oxidation.

Year (0 = SU)	Investment or salvage value	Working capital	Start-up capital	Net manufacturing cost [b]	Total cash flow	Discount factor
**-2**	−2,837,372	-	-	-	−2,837,372	1.21
**-1**	−21,280,288	-	-	-	−21,280,288	1.10
**0**	−11,349,487	−1,773,357	-1,418,686	-	−14,541,530	1.00
**1**	-	-	-	-4,275,410	-4,275,410	0.91
**2**	-	-	-	-5,704,454	-5,704,454	0.83
**3**	-	-	-	-7,946,779	-7,946,779	0.75
**4**	-	-	-	-9,323,542	-9,323,542	0.68
**5**	-	-	-	-9,323,542	-9,323,542	0.62
**6**	-	-	-	-9,323,542	-9,323,542	0.56
**7**	-	-	-	-9,323,542	-9,323,542	0.51
**8**	-	-	-	-9,323,542	-9,323,542	0.47
**9**	-	-	-	-9,323,542	-9,323,542	0.42
**10**	1,418,686 [a]	1,773,357	-	-9,323,542	-6,131,499	0.39

Year (0=SU)	Revenues [c] (R)	Operation cost (C)					Depreciation
		Raw materials	Utilities	Fixed operating costs	Other operation costs	Total operation costs	
**-2**	0	0	0	0	0	0	0
**-1**	0	0	0	0	0	0	0
**0**	0	0	0	0	0	0	0
**1**	4,705,420	5,429,313	5,630,400	1,995,830	137,200	13,192,743	3,546,715
**2**	6,796,718	7,842,341	7,507,200	1,995,830	137,200	17,482,571	3,546,715
**3**	8,888,016	10,255,369	10,635,200	1,995,830	137,200	23,023,599	3,546,715
**4**	10,456,490	12,065,140	12,512,000	1,995,830	137,200	26,710,170	3,546,715
**5**	10,456,490	12,065,140	12,512,000	1,995,830	137,200	26,710,170	3,546,715
**6**	10,456,490	12,065,140	12,512,000	1,995,830	137,200	26,710,170	3,546,715
**7**	10,456,490	12,065,140	12,512,000	1,995,830	137,200	26,710,170	3,546,715
**8**	10,456,490	12,065,140	12,512,000	1,995,830	137,200	26,710,170	3,546,715
**9**	10,456,490	12,065,140	12,512,000	1,995,830	137,200	26,710,170	3,546,715
**10**	10,456,490	12,065,140	12,512,000	1,995,830	137,200	26,710,170	3,546,715

[a] 4% of the Fixed Capital

[b] without the revenues of GRA

[c] without the revenues of GRA

Supplementary Table 15 | Discounted cash flow for the non-electrocatalytic glucose oxidation.

Year (0 = SU)	Investment or salvage value	Working capital	Start-up capital	Net manufacturing cost [b]	Total cash flow	Discount factor
**-2**	−4,328,547	-	-	-	−4,328,547	1.21
**-1**	−32,464,105	-	-	-	−32,464,105	1.10
**0**	−17,314,189	−2,705,342	-2,164,272	-	−22,183,803	1.00
**1**	-	-	-	-7,674,209	-7,674,209	0.91
**2**	-	-	-	-11,080,608	-11,080,608	0.83
**3**	-	-	-	-14,487,007	-14,487,007	0.75
**4**	-	-	-	-17,041,807	-17,041,807	0.68
**5**	-	-	-	-17,041,807	-17,041,807	0.62
**6**	-	-	-	-17,041,807	-17,041,807	0.56
**7**	-	-	-	-17,041,807	-17,041,807	0.51
**8**	-	-	-	-17,041,807	-17,041,807	0.47
**9**	-	-	-	-17,041,807	-17,041,807	0.42
**10**	2,164,274 [a]	2,705,342	-	-17,041,807	-12,172,191	0.39

Year (0=SU)	Revenues [c] (R)	Operation cost (C)					Depreciation
		Raw materials	Utilities	Fixed operating costs	Other operation costs	Total operation costs	
**-2**	0	0	0	0	0	0	0
**-1**	0	0	0	0	0	0	0
**0**	0	0	0	0	0	0	0
**1**	277,200	8,690,071	3,378,510	2,571,650	356,890	14,997,121	5,410,684
**2**	400,400	12,552,325	4,880,070	2,571,650	356,890	20,360,935	5,410,684
**3**	523,600	16,414,579	6,381,630	2,571,650	356,890	25,724,749	5,410,684
**4**	616,000	19,311,270	7,507,800	2,571,650	356,890	29,747,610	5,410,684
**5**	616,000	19,311,270	7,507,800	2,571,650	356,890	29,747,610	5,410,684
**6**	616,000	19,311,270	7,507,800	2,571,650	356,890	29,747,610	5,410,684
**7**	616,000	19,311,270	7,507,800	2,571,650	356,890	29,747,610	5,410,684
**8**	616,000	19,311,270	7,507,800	2,571,650	356,890	29,747,610	5,410,684
**9**	616,000	19,311,270	7,507,800	2,571,650	356,890	29,747,610	5,410,684
**10**	616,000	19,311,270	7,507,800	2,571,650	356,890	29,747,610	5,410,684

[a] 4% of the Fixed Capital

[b] without the revenues of GRA

[c] without the revenues of GRA

Supplementary Notes 1. Chemicals and materials

All chemicals used were analytical grade and used without further purification. Glucose, Gluconic acid (GNA), Glucaric acid (GRA), iron(III) chloride hexahydrate ($FeCl_3$•$6H_2O$), urea, and ethanol were purchased from Shanghai Sinopharm Chemical Reagent Co., China. Nickel foam (NF) with a thickness of 1.5 mm was obtained from Suzhou Jiashide Metal Foam Co., China. Before use, the NF was cut down into 2×2 cm^2 pieces, and cleaned with hydrochloric acid with ultrasonication for 30 min to remove the surface oxides, then washed sequentially with water and ethanol several times, and stored in ethanol for further use.

Supplementary Methods 1. Characterizations of the catalysts

The X-ray diffraction (XRD) analysis was performed with a MXPAHF X-ray diffractometer (Rigaku Co., Japan) equipped with a nickel-filtered Cu Kα radiation source (30 kV/160 mA, λ= 0.154 nm). The samples were scanned from 20° to 80° at a scan rate (2θ) of 0.02^o/s. The diffraction peaks were attributed to the corresponding crystalline phases with reference to the powder diffraction files from the Joint Committee on Power Diffraction Standards (JCPDS). The morphology of the samples was imaged by a scanning electron microscope (SEM, Sirion 200, FEI Co., USA). The elemental mapping in selected areas of the SEM image was conducted with an energy dispersive X-ray spectroscopy (EDS) equipped with a Thermo-Fisher EDS detector at 15 kV. X-ray photoelectron spectroscopy (XPS) analysis was performed via an X-ray photoelectron spectrometer (ESCALAB250, Thermo-VG Scientific Inc.,

UK) using monochromatized Al Kα radiation (1486.92 eV). The active mass loading of the electrocatalysts was described by their Fe contents, which were analyzed by inductively coupled plasma atomic emission spectroscopy (ICP-AES, Optima 7300DV, PerkinElmer Corporation, USA) after digestion with HNO_3.

Supplementary Methods 2. Quantification analysis of the reaction products

The concentration changes of glucose and its oxidation products in electrolysis process were monitored by high performance liquid chromatography (HPLC, LC-20AD, Shimadzu Co., Japan) equipped with a refractive index detector. Sulfuric acid (H_2SO_4, 5 mM) was used as the mobile phase with a constant flow rate of 0.5 mL/min. In each analysis, 100 μL of the electrolyte solution was withdrawn from the electrochemical cell in chronoamperometry test and diluted to 1.0 mL with 0.2 M of H_2SO_4 solution to adjust the pH below 7.0, then 10 μL of the diluted solution was injected directly into a BioRad Aminex 87H column with a column temperature of 60 ^oC. The identification of glucose and its oxidation products was achieved by comparing their retention times in the chromatograms with those of the standard solution, and their concentrations were determined from calibration curves made by applying standard solutions with known concentrations. The conversion of glucose ($\eta_{glucose}$) and yields of its oxidation products (Y_P) were calculated using the following equations (Eqs. 1 and 2):

$$\eta_{glucose} = (1 - C_{glucose}/C_{0\text{-}glucose}) \times 100\% \quad (1)$$

$$Y_P = C_P/C_{0\text{-}glucose} \times 100\% \quad (2)$$

where $C_{0\text{-}glucose}$ and $C_{glucose}$ are the initial glucose concentration and the concentrations of glucose at different reaction times, respectively, and C_p is the concentration of glucose oxidation products (GNA and GRA) at different reaction times. The Faradaic efficiency (FE) toward GNA and GRA production was calculated from the total amount of charge Q (in units of coulombs) passed through the electrochemical cell and the total amount N (in units of moles) of GNA and GRA production. $Q = J \times S \times t$, where J (A/cm^2) is the current density at a specific applied potential, S is the electrode area (cm^2) and t is the reaction time (seconds). Since 2 electrons are required to convert one glucose molecule into GNA and 6 electrons are required to converted glucose into GRA, the Faradaic efficiency can be calculated as follows (Eq. 3):

$$FE = 2F \times N_{(GNA)} + 6F \times N_{(GRA)}/Q = 2F \times N_{(GNA)} + 6F \times N_{(GRA)}/(J \times S \times t) \quad (3)$$

where F is the Faraday constant (F= 96485 C/mol).

The products and intermediates of glucose electrolysis after 6-h reaction were characterized by 2D HSQC nuclear magnetic resonance (NMR). The alkaline solution obtained from the electrolysis cell was first neutralized with HCl, then water was evaporated under reduced pressure at 50 ℃ and the obtained solid was re-dissolved with ethyl acetate (the formed KCl could not dissolve into ethyl acetate). The ethyl acetate solution was then evaporated under reduced pressure at 30 ℃, and the obtained solid was dissolved with DMSO-d6 to form a solution for NMR analysis. The 2D HSQC NMR experiments were carried out on a MHz spectrometer (AVANCE AV III 400, Bruker Co., USA).

The turnover frequency (TOF) values of the catalyst based on the collected LSV

data using a widely accepted equation listed as follows (Eq. 4):[33, 34]

$$TOF=j*s/(n*F*m) \qquad (4)$$

where j is the current density obtained at mentioned potentials, s is the surface area of the electrode with catalyst loading (1.0 cm^2), F is the Faraday efficiency (96,485 C/mol) and m is the number of moles of active sites deposited onto the electrodes, assuming all the metal sites were involved in the electrochemical reaction, n is the electrons transfer during the reaction (n is 6 for glucose oxidation, 4 for oxygen evolution, and 2 for hydrogen evolution). The TOF values were calculated with this equation and are presented in Supplementary Table 5.

Supplementary Methods 3. Calculation of the cost and benefit for the glucose electrolysis

First, the overall reaction occurring in the electrolysis process can be expressed as follows (Eqs. 5 and 6):

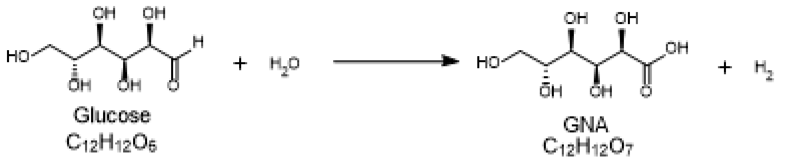

(5)

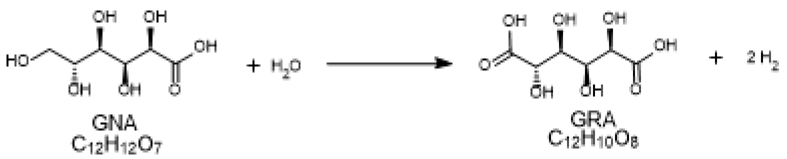

(6)

To simplify the calculation process, we assume that the total conversion of glucose is 90%, and the selectivity to GNA and GRA is 20% and 80%, respectively. According to Eqs S1 and S2, when 1 mol (0.181 kg) of glucose is consumed, 0.18 mol (0.035 kg) of GNA and 0.72 mol (0.151 kg) of GRA are produced in the anode.

Meanwhile, 2.34 mol (0.00468 kg) of H_2 is produced in the cathode. Making a mathematical transformation, we can know that, to produce every 1 kg of H_2, 38.67 kg of glucose is consumed, co-producing 7.48 kg of GNA and 32.26 kg of GRA.

Supplementary Methods 4. ASPEN simulations of the electrocatalytic glucose oxidation and the non- electrocatalytic glucose oxidation

In this section, two different processes, i.e., electrocatalytic glucose oxidation and non-electrocatalytic glucose oxidation, were simulated using the ASPEN Plus software to highlight the advantages of the electrocatalytic glucose oxidation. The electrocatalytic glucose oxidation process consists of two main processing steps, i.e., GRA production, and GRA separation and purification. A mixture of 0.1 M glucose in 1 M KOH solution was sent to the electrolysis cell, and the electrolysis time was set as 18 h. To simplify the simulation process, we assume that the total production amount of GRA is 1000 tons per year, the glucose conversion is 90%, and the selectivity to GNA and GRA is 20% and 80%, respectively. Thus, the glucose consumption is about 1200 tons per year, co-producing 230 tons of GNA in the anode, and 31 ton of H_2 in the cathode. Prior to the recovery of GRA and GNA, the KOH present in the liquid mixture is neutralized with H_2SO_4 (10 wt.%, 3550 tons per year). Then, the neutralized mixture is shifted to distillation column to remove the solvent (water), and the remaining concentrated liquid is then sent to crystallizers to recover the target chemicals (GRA, GNA, and K_2SO_4). The obtained solid mixture is then separated and purified via two-stage re-crystallization. The abovementioned process is

numerically simulated using the ASPEN Plus Process Simulator and the associated data for the electrocatalytic glucose process are presented in Supplementary Table 6.

The non-electrocatalytic glucose oxidation follows a similar process to that of electrocatalytic oxidation, but uses HNO_3 as an oxidant. A gas−liquid absorber is employed to absorb the unreacted HNO_3 and the resulting NO_x from the reaction mixture. Supplementary Table 7 lists the related data for the non-electrocatalytic oxidation process.

Supplementary references

1. Shuai, W. *et al.* Ultrastable In-Plane 1T–2H MoS2 Heterostructures for Enhanced Hydrogen Evolution Reaction. *Adv. Energy Mater.* **8**, 1801345 (2018).
2. Yiqiang, S. *et al.* Strong Electronic Interaction in Dual-Cation-Incorporated NiSe2 Nanosheets with Lattice Distortion for Highly Efficient Overall Water Splitting. *Adv. Mater.* **30**, 1802121 (2018).
3. Fu, Q. *et al.* Skutterudite-Type Ternary Co1–xNixP3 Nanoneedle Array Electrocatalysts for Enhanced Hydrogen and Oxygen Evolution. *ACS Energy Lett.* **3**, 1744-1752 (2018).
4. Yichao, H. *et al.* Fine Tuning Electronic Structure of Catalysts through Atomic Engineering for Enhanced Hydrogen Evolution. *Adv. Energy Mater.* **8**, 1800789 (2018).
5. Guowei, L. *et al.* Carbon-Tailored Semimetal MoP as an Efficient Hydrogen Evolution Electrocatalyst in Both Alkaline and Acid Media. *Adv. Energy Mater.* **8**, 1801258 (2018).
6. Zheng, Y.-R. *et al.* Doping-induced structural phase transition in cobalt diselenide enables enhanced hydrogen evolution catalysis. *Nature Commun.* **9**, 2533 (2018).
7. Masa, J. *et al.* Low overpotential water splitting using cobalt–cobalt phosphide nanoparticles supported on nickel foam. *ACS Energy Lett.* **1**, 1192-1198 (2016).
8. Zhang, B. *et al.* Iron–nickel nitride nanostructures in situ grown on surface-redox-etching nickel foam: Efficient and ultrasustainable electrocatalysts for overall water splitting. *Chem. Mater.* **28**, 6934-6941 (2016).
9. Zhang, X. *et al.* Facile synthesis of nickel–iron/nanocarbon hybrids as advanced electrocatalysts for efficient water splitting. *ACS Catal.* **6**, 580-588 (2015).
10. Mishra, I.K. *et al.* Hierarchical CoP/Ni5P4/CoP microsheet arrays as a robust pH-universal electrocatalyst for efficient hydrogen generation. *Energy Environ.*

Sci. **11**, 2246-2252 (2018).

11. Menezes, P.W. *et al.* A structurally versatile nickel phosphite acting as a robust bifunctional electrocatalyst for overall water splitting. *Energy Environ. Sci.* **11**, 1287-1298 (2018).
12. Hu, E. *et al.* Construction of hierarchical Ni–Co–P hollow nanobricks with oriented nanosheets for efficient overall water splitting. *Energy Environ. Sci.* **11**, 872-880 (2018).
13. Miao, R. *et al.* Mesoporous iron sulfide for highly efficient electrocatalytic hydrogen evolution. *J. Am. Chem. Soc.* **139**, 13604-13607 (2017).
14. Feng, J.-X., Wu, J.-Q., Tong, Y.-X. & Li, G.-R. Efficient hydrogen evolution on Cu nanodots-decorated Ni3S2 nanotubes by optimizing atomic hydrogen adsorption and desorption. *J. Am. Chem. Soc.* **140**, 610-617 (2018).
15. Panda, C. *et al.* From a Molecular 2Fe-2Se Precursor to a Highly Efficient Iron Diselenide Electrocatalyst for Overall Water Splitting. *Angew. Chem. Int. Ed.* **56**, 10506-10510 (2017).
16. Zhang, Y. *et al.* Ultrafine Metal Nanoparticles/N-Doped Porous Carbon Hybrids Coated on Carbon Fibers as Flexible and Binder-Free Water Splitting Catalysts. *Adv. Energy Mater.* **7**, 1700220 (2017).
17. Dai, L. *et al.* Electrochemical Partial Reforming of Ethanol into Ethyl Acetate Using Ultrathin Co3O4 Nanosheets as a Highly Selective Anode Catalyst. *ACS Cent. Sci.* **2**, 538-544 (2016).
18. Chen, G.-F., Luo, Y., Ding, L.-X. & Wang, H. Low-Voltage Electrolytic Hydrogen Production Derived from Efficient Water and Ethanol Oxidation on Fluorine-Modified FeOOH Anode. *ACS Catal.* **8**, 526-530 (2017).
19. Chen, S., Duan, J., Vasileff, A. & Qiao, S.Z. Size Fractionation of Two-Dimensional Sub-Nanometer Thin Manganese Dioxide Crystals towards Superior Urea Electrocatalytic Conversion. *Angew. Chem. Int. Ed.* **55**, 3804-3808 (2016).
20. Yu, Z.-Y. *et al.* Ni-Mo-O nanorod-derived composite catalysts for efficient

alkaline water-to-hydrogen conversion via urea electrolysis. *Energy Environ. Sci.* **11**, 1890-1897 (2018).

21. Xiao, C., Li, S., Zhang, X. & MacFarlane, D.R. MnO2/MnCo2O4/Ni heterostructure with quadruple hierarchy: a bifunctional electrode architecture for overall urea oxidation. *J. Mater. Chem. A* **5**, 7825-7832 (2017).
22. Liu, D. *et al.* High-performance urea electrolysis towards less energy-intensive electrochemical hydrogen production using a bifunctional catalyst electrode. *J. Mater. Chem. A* **5**, 3208-3213 (2017).
23. Liu, Q. *et al.* A porous Ni3N nanosheet array as a high-performance non-noble-metal catalyst for urea-assisted electrochemical hydrogen production. *Inorg. Chem. Fron.* **4**, 1120-1124 (2017).
24. Jiang, N., You, B., Boonstra, R., Terrero Rodriguez, I.M. & Sun, Y. Integrating Electrocatalytic 5-Hydroxymethylfurfural Oxidation and Hydrogen Production via Co–P-Derived Electrocatalysts. *ACS Energy Lett.* **1**, 386-390 (2016).
25. You, B., Liu, X., Jiang, N. & Sun, Y. A General Strategy for Decoupled Hydrogen Production from Water Splitting by Integrating Oxidative Biomass Valorization. *J. Am. Chem. Soc.* **138**, 13639-13646 (2016).
26. You, B., Jiang, N., Liu, X. & Sun, Y. Simultaneous H2 Generation and Biomass Upgrading in Water by an Efficient Noble-Metal-Free Bifunctional Electrocatalyst. *Angew. Chem. Int. Ed.* **55**, 9913-9917 (2016).
27. Jiang, N. *et al.* Electrocatalysis of Furfural Oxidation Coupled with H2 Evolution via Nickel-Based Electrocatalysts in Water. *ChemNanoMat* **3**, 491-495 (2017).
28. You, B., Liu, X., Liu, X. & Sun, Y. Efficient H2 Evolution Coupled with Oxidative Refining of Alcohols via A Hierarchically Porous Nickel Bifunctional Electrocatalyst. *ACS Catal.* **7**, 4564-4570 (2017).
29. Jian, Z. *et al.* Hierarchical Porous NC@CuCo Nitride Nanosheet Networks: Highly Efficient Bifunctional Electrocatalyst for Overall Water Splitting and Selective Electrooxidation of Benzyl Alcohol. *Adv. Func. Mater.* **27**, 1704169

(2017).

30. Du, P., Zhang, J., Liu, Y. & Huang, M. Hydrogen generation from catalytic glucose oxidation by Fe-based electrocatalysts. *Electrochem. Commun.* **83**, 11-15 (2017).

31. Wu, T. *et al.* Vapor-phase hydrothermal growth of single crystalline NiS_2 nanostructure film on carbon fiber cloth for electrocatalytic oxidation of alcohols to ketones and simultaneous H_2 evolution. *Nano Res.* **11**, 1004-1017 (2018).

32. Lyu, C. *et al.* Homologous Co_3O_4-CoP nanowires grown on carbon cloth as a high-performance electrode pair for triclosan degradation and hydrogen evolution. *Mater. Chem Fron.* **2**, 323-330 (2018).

33. Lu et al., Electrodeposition of hierarchically structured three-dimensional nickel–iron electrodes for efficient oxygen evolution at high current densities. Nat. Commun. 6, 6616 (2015);

34. Gong et al. An advanced Ni–Fe layered double hydroxide electrocatalyst for water oxidation. J. Am. Chem. Soc. 135, 8452-8455 (2013)
